# Using Microsatellites to Understand the Physical Distribution of Recombination on Soybean Chromosomes

**DOI:** 10.1371/journal.pone.0022306

**Published:** 2011-07-20

**Authors:** Alina Ott, Brian Trautschold, Devinder Sandhu

**Affiliations:** Department of Biology, University of Wisconsin-Stevens Point, Stevens Point, Wisconsin, United States of America; University of Umeå, Sweden

## Abstract

Soybean is a major crop that is an important source of oil and proteins. A number of genetic linkage maps have been developed in soybean. Specifically, hundreds of simple sequence repeat (SSR) markers have been developed and mapped. Recent sequencing of the soybean genome resulted in the generation of vast amounts of genetic information. The objectives of this investigation were to use SSR markers in developing a connection between genetic and physical maps and to determine the physical distribution of recombination on soybean chromosomes. A total of 2,188 SSRs were used for sequence-based physical localization on soybean chromosomes. Linkage information was used from different maps to create an integrated genetic map. Comparison of the integrated genetic linkage maps and sequence based physical maps revealed that the distal 25% of each chromosome was the most marker-dense, containing an average of 47.4% of the SSR markers and 50.2% of the genes. The proximal 25% of each chromosome contained only 7.4% of the markers and 6.7% of the genes. At the whole genome level, the marker density and gene density showed a high correlation (R^2^) of 0.64 and 0.83, respectively with the physical distance from the centromere. Recombination followed a similar pattern with comparisons indicating that recombination is high in telomeric regions, though the correlation between crossover frequency and distance from the centromeres is low (R^2^ = 0.21). Most of the centromeric regions were low in recombination. The crossover frequency for the entire soybean genome was 7.2%, with extremes much higher and lower than average. The number of recombination hotspots varied from 1 to 12 per chromosome. A high correlation of 0.83 between the distribution of SSR markers and genes suggested close association of SSRs with genes. The knowledge of distribution of recombination on chromosomes may be applied in characterizing and targeting genes.

## Introduction

Soybean {*Glycine max* (L.) Merr.} is an important crop globally. Soybeans are a key source of protein and oil for human consumption, and are also vital for animal feed and other raw materials. Due to the importance of the soybean as a major crop, ongoing genetic research has made great advances in comprehending the soybean genome. Genetic maps give an understanding of the genetic make-up of a genome and allow for localization of genes of interest through genetic linkage analysis with maed markers [1]. Several marker-based genetic linkage maps have been developed for soybean, the first of which was developed in 1990 [2]. Within the next few years various types of markers, including restriction fragment length polymerase (RFLP) [3], single nucleotide polymorphism (SNP) [4], randomly amplified polymorphic DNA (RAPD) [5], amplified fragment length polymerase (AFLP) [6], and simple sequence repeats (SSR) [7], were used to develop genetic maps in soybean.

SSR markers, also referred to as microsatellites, are an especially useful marker type. They are inherited in a co-dominant manner, exhibit high levels of polymorphism, are spread throughout the genome, undergo polymerase chain reactions (PCR) easily, and can be analyzed with gel electrophoresis [7], [8]. Because of their ease and reliability, SSR markers have become an important method of genetically mapping soybean genes. Over the years thousands of SSR markers were developed from both expressed sequence tags (ESTs) and genomic DNA, adding to the number of available SSRs [9], [10], [11], [12], [13], [14], [15]. Recently, approximately 33,000 putative SSR markers have been developed from the soybean genome sequence and some of them have been mapped [15].

Marker distribution is often associated with gene distribution. Supporting this association, a study of microsatellites in *Arabidopsis*, rice, maize, wheat and soybean clearly demonstrated that microsatellite distribution was much higher in gene-rich, single copy regions as compared to repetitive sections of the genome [16]. In addition, a comparison of RFLP and SSR markers in soybean found that RFLPs tend to be more closely associated with gene-rich regions; however, SSR loci were closely associated with actual genic sequences [17].

Many major crops have been found to have clustered genes in gene-rich regions on chromosomes, which follow the patterns of clustered markers [18], [19], [20], [21]. In wheat, the major gene-rich regions were only found in the distal 35% of the chromosome arms [19]. While some gene-rich areas do exist in the proximal portions of the chromosomes, almost no recombination occurs in these areas due to the proximity of the centromere, making marker-based mapping ineffective for the genes present in these regions [18]. ESTs in maize are predominately associated with the distal region, with 36% of the ESTs being observed in the distal 20% of the chromosome arms and only 7% in the proximal 20% [22]. Barley and rice, other members of the family *Gramineae* along with maize, show clustered patterns of gene distribution [21].

Distribution of recombination on chromosomes follows a similar pattern to gene distribution. Using physical maps, recombination was found to be suppressed near the rice centromeres [23] with the same being true in corn [24]. A detailed study comparing genetic and physical maps of chromosome 3B in wheat confirmed that most recombination is found in the telomeric regions with only three crossovers identified in the proximal centromeric regions [25]. Recombination was several folds higher in the telomeric regions as compared to the centromeric regions [26]. In barley, a 1 Mb segment in a gene-rich part of a chromosome may have as much as a 10-fold difference in recombination, with even higher differences of 20-fold in humans and rice [27].

The soybean genome was recently sequenced, allowing for physical comparisons of gene and marker distribution [28]. Together, marker-based linkage mapping and sequence based physical mapping allow for an even more in-depth look at the soybean genome. Knowledge of marker distribution and recombination grants a powerful tool for characterizing and tagging genes. Being able to identify regions of high recombination but low marker distribution allows for a more thorough analysis of genes of interest and their actual locations. Knowing the physical distance between markers facilitates fine-mapping of genes that were previously impossible to locate and clone. With genome sequencing, the physical location of the nearest markers can be used to find the number and function of potential target genes.

The objectives of this investigation were to use SSR markers in developing a connection between genetic and physical maps and to determine the physical distribution of recombination on soybean chromosomes.

## Materials and Methods

### Markers

A total of 1028 GMES (*Glycine max* EST SSR) markers, 168 CSSR (Chiba University Simple Sequence Repeat) markers, 330 Sat markers, 594 Satt markers, 28 Sct/Sctt markers, and 40 other SSR markers were physically located onto soybean chromosomes using the Phytozome database (http://www.phytozome.net/) [9], [10], [11], [12], [13], [14]. GMES markers are SSR markers derived from non-redundant ESTs [12]. CSSR markers are SSR markers developed at Chiba University, Japan [10], [13]. Sat and Satt markers contain AT and ATT repeats respectively [9], [11]. Sct and Sctt markers contain CT and CTT repeats respectively [9], [11].

### Searching Phytozome

An online database, Phytozome, provides a chromosome-scale sequence that is searchable (http://www.phytozome.net/soybean). The SSR primer sequences were searched in the Phytozome database and the base pair locations of the beginning of the forward primer and end of the reverse primer were identified (Figure S1). Many of the Sat and Satt markers and some of the other markers were already located within the sequence and were therefore searchable by ID number instead of primer sequence [15]. We located 1185 additional markers: 909 GMES, 141 CSSR markers, 70 Satt markers, 41 Sat markers, 5 Sct, 3 Sctt markers, and 16 other SSR markers. For the markers that were developed from ESTs and where only one or neither primer was located, we searched for the sequence ID in the NCIB database (http://www.ncbi.nlm.nih.gov). We then used the provided EST sequence to find the base pair location of the marker. Some markers were not found using the ID numbers or sequences, and these markers were not included in the study. Markers for which more than one location was found on the known chromosome were also not included to prevent false placement. In cases where only one primer was matched in the database, the marker was included.

### Creating Genetic and Physical Maps

After each marker was located, the distance between each marker's start and ending sequence location was averaged to obtain an average physical location. The average physical locations were entered into the program “MapChart” to create a physical map of each chromosome (Figure S1) [29].

To create the integrated linkage maps we combined information from three recombination maps [11], [13], [14]. Due to the differences in genetic location of the same markers on different maps, integration of the three maps was required before a genetic map was created. We identified the common markers between all three maps and determined the distances between these markers on each map. These distances were then compared to determine proportionate distances between different maps for a particular chromosomal region. The calculated ratios were used to place all the unique markers from each map on the same scale. For telomeric regions where there were no common flanking markers, the ratio from the closest flanked region was used to place the unique markers. The integrated recombination values were then entered into “MapChart” to create a genetic map of each chromosome (Figure S1) [29].

### Distribution of Markers and Genes

**Figure 1:**
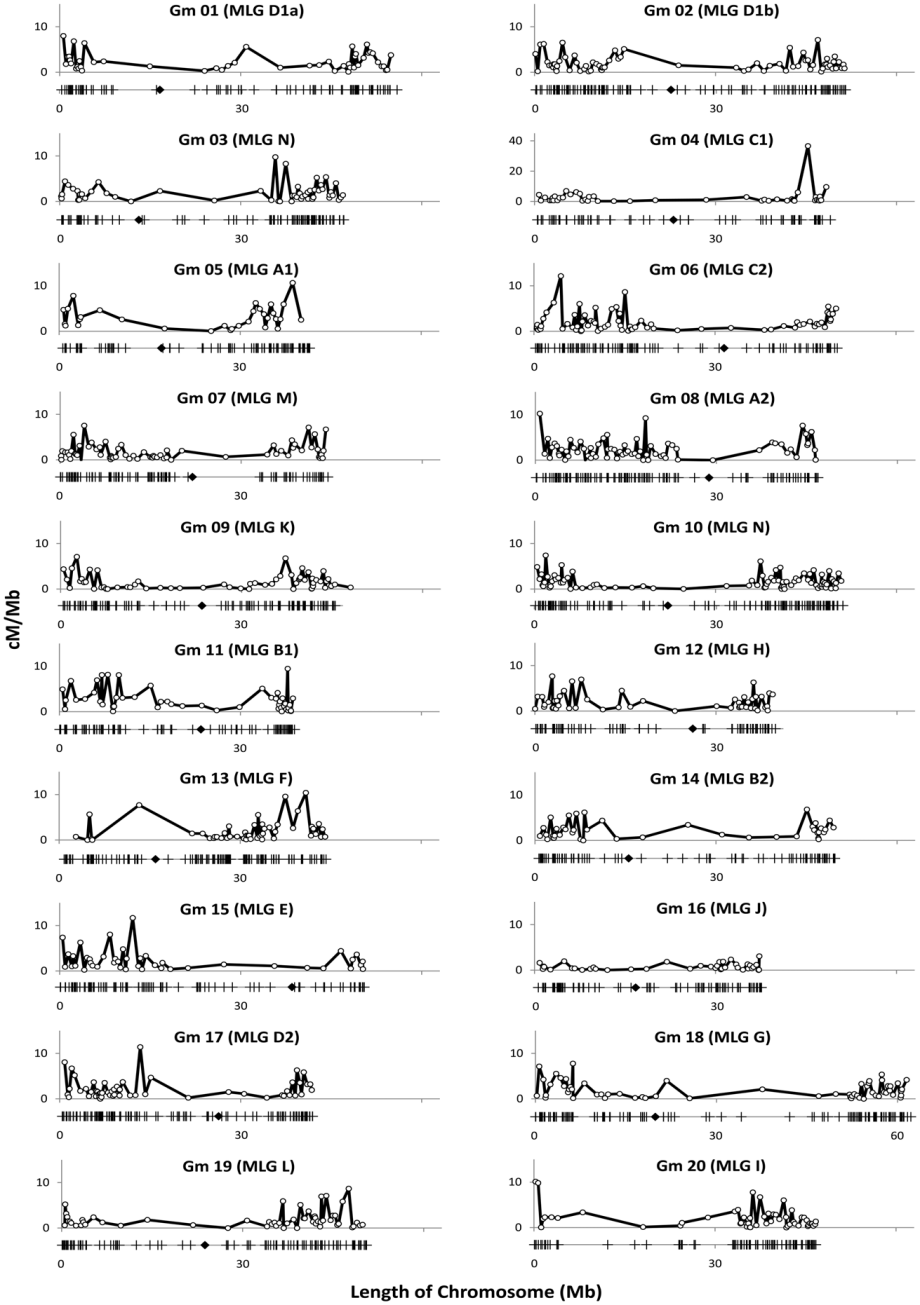
Physical distribution of recombination and SSR markers on twenty soybean chromosomes. The Y-axis represents cM/Mb and the X-axis represents length of the chromosome in Mb. Vertical marks (I) on the line below the X-axis represent the location of the SSR markers. Diamond (♦) represents the location of the centromere.

We graphed the average physical locations of all the markers on a scatter plot to show the distribution of SSR markers throughout the chromosome (Figure 1). In addition, we determined the location of the centromere of each chromosome using Phytozome and included them to compare the location of the centromere with the distribution of markers. The recombination between each set of markers was determined from the integrated genetic maps and graphed on a scatter plot versus the average physical location. The graphs were aligned to highlight the distribution of markers and location of the centromeres compared to areas of high and low recombination (Figure 1).

**Figure 2:**
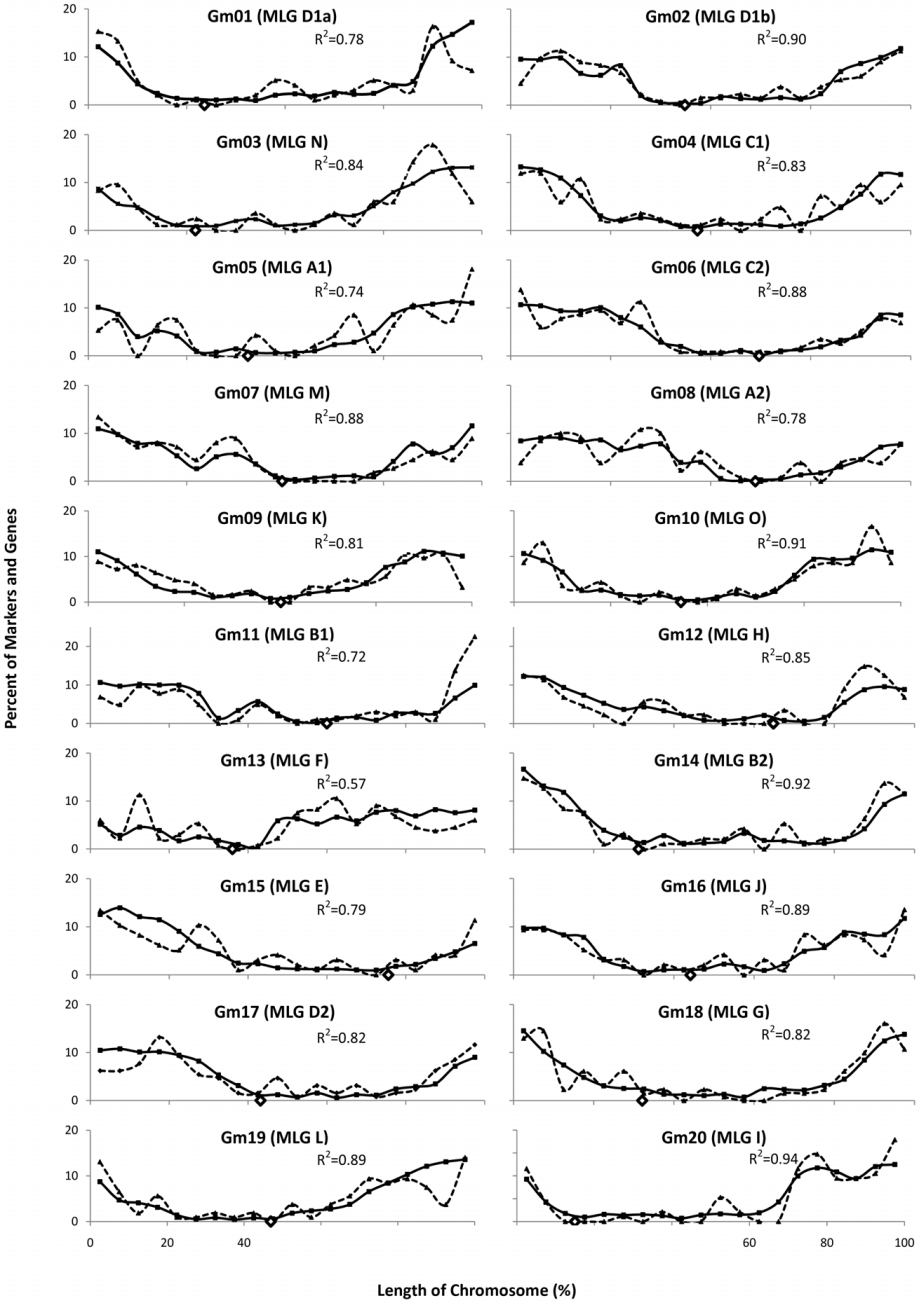
Distribution of predicted genes and SSR markers on twenty soybean chromosomes. The Y-axis represents percent genes or markers present in each of the 20 segments per chromosome. The X-axis represents length of the chromosome in Mb. Solid lines indicate gene distribution. Dashed lines represent marker distribution.

We also graphed the percent of genes and markers in factions of the chromosomes to show the correlation between SSR markers and genes (Figure 2). We used the physical locations of predicted open reading frames from Phytozome to identify the location of all 46,430 protein coding genes [28]. We then divided the total length of the chromosome into 20 sections and calculated the number of genes in each 5% chromosomal region. The number of genes in each region was then converted to a percent of the total number of genes for the chromosome and graphed. The same was done for the number of markers in each 5% chromosomal region.

Using the densities calculated for Figure 2, we determined the correlation between the density of markers and their distance from the centromere along with the density of genes and their distance from the centromere (Table 1). We also determined the correlation between marker and gene density in each 5% chromosomal region (Table 1, Figure 2).

**Table 1:** Distribution of genes and SSR markers on soybean chromosomes.

Chromosome #	MLG	Percent SSRs in Distal 25%	Percent SSRs in Proximal 25%	Percent Genes in Distal 25%	Percent Genes in Proximal 25%	Correlation of Marker Density with Distance from Centromere	Correlation of Gene Density with Distance from Centromere	Correlation of Marker and Gene Distribution
Gm01	D1a	59.18	6.12	63.61	5.92	0.44	0.68	0.779
Gm02	D1b	43.61	6.02	48.38	4.10	0.62	0.72	0.898
Gm03	N	57.14	7.14	55.66	7.28	0.63	0.81	0.844
Gm04	C1	46.43	7.14	57.11	6.27	0.73	0.80	0.832
Gm05	A1	45.74	5.32	51.53	4.14	0.75	0.91	0.737
Gm06	C2	43.97	4.31	47.49	3.53	0.85	0.95	0.881
Gm07	M	41.07	10.71	45.26	9.30	0.56	0.78	0.884
Gm08	A2	35.11	9.16	41.40	4.66	0.57	0.89	0.780
Gm09	K	41.60	7.20	50.06	7.32	0.81	0.92	0.815
Gm10	O	53.96	5.76	51.87	5.28	0.84	0.92	0.906
Gm11	B1	64.71	6.86	54.08	6.11	0.59	0.88	0.722
Gm12	H	43.18	4.55	47.74	5.60	0.59	0.86	0.851
Gm13	F	22.56	12.03	31.39	13.42	0.35	0.86	0.574
Gm14	B2	55.79	5.26	50.00	9.57	0.35	0.92	0.919
Gm15	E	49.48	7.22	54.86	6.66	0.76	0.90	0.794
Gm16	J	42.71	9.38	46.48	7.53	0.78	0.88	0.889
Gm17	D2	41.86	11.63	47.85	4.88	0.65	0.76	0.817
Gm18	G	61.83	12.21	57.10	9.12	0.43	0.50	0.892
Gm19	L	42.06	7.48	48.32	6.20	0.76	0.91	0.772
Gm20	I	56.84	2.11	53.94	7.23	0.65	0.72	0.936
Genome Average		47.44	7.38	50.21	6.71	0.64	0.83	0.826

From the locations of markers and genes, we determined the percent of the total genes and markers located in the proximal and distal 25% of each chromosome (Table 1). The percent of genes and markers in the proximal 25% of each chromosome arm were added together to obtain the proximal 25% for the entire chromosome, giving a number unbiased by unequal chromosome arm length. The same was done for the distal 25%.

### Crossover Frequency

Crossover frequency (CF) was determined by calculating the genetic distance (cM) between two markers divided by the physical distance (MB) between the same two markers. CF was calculated for each set of markers. Markers where the physical and genetic order did not match were not included in these calculations. Hotspots were considered areas between two markers where CF was greater than twice the genome average.

For the average CF in the proximal and distal 25% of the chromosomes, we first determined the physical location marking 25% of the proximal and distal region of each chromosome arm. CF values in both distal regions (for both the chromosome arms) of the same chromosome were averaged together to give 25% of the entire chromosome's distal region. The same calculations were done for the proximal regions of each chromosome. The correlation between CF and distance from the centromere was calculated.

## Results

### Physical distribution of SSRs and genes on soybean chromosomes

In this investigation we used 2188 SSR markers with an average of 109 markers per chromosome. Chromosome Gm10 contained the maximum number of 139 markers and chromosome Gm03 and Gm04 each contained the minimum number of 84 markers. The average distance between markers for the whole genome was 446,124 base pairs (bp). At the chromosomal level, the average distance between markers was largest for chromosome Gm04, where on average markers were 585,370 bp apart. Chromosome Gm17 showed the smallest average distance between markers, where on average markers were 323,393 bp apart.

SSR markers were not uniformly distributed on soybean chromosomes. For most of the chromosomes, some regions were marker dense and others were very low in marker density (Figure 1). A number of chromosomes showed several marker clusters throughout the chromosome. For example, chromosome Gm04 showed at least four clusters near the telomere of the short arm and four clusters near the telomere of the long arm (Figure 1). Chromosome Gm02 showed a linear increase in the number of markers towards both telomeres. Chromosome Gm11 has two large marker clusters near the telomere of the short arm and several small marker clusters scattered all over the long arm. In general, more markers clustered toward the telomeres with a region of relatively few markers close to the centromere. However, chromosomes like Gm05, Gm06, Gm11, Gm15 and Gm20 had a much higher density of markers on one chromosome arm than the other, likely due to their centromeres being off-center. In addition, Gm02, Gm09, Gm10, and Gm13 had much smaller central regions lacking in markers and more markers spread throughout these proximal areas compared to the other chromosomes.

To study the distribution of markers in relation to the centromeres, centromeric repeat sequences were used to localize centromeric regions on each of the soybean chromosomes [28]. The density of SSR markers was much higher on the distal ends of the chromosomes than in the regions proximal to the centromeres. The distal 25% of the chromosomes contained 47.4% of the SSRs while the proximal 25% contained only 7.4% of the SSRs (Table 1). The distal ends ranged from 22.6% of the SSRs for chromosome Gm13 to 64.7% of the SSRs for Gm11. The proximal regions ranged from 2.1% of the SSRs for chromosome Gm20 to 12.2% of the SSRs for Gm18 (Table 1). Though not uniformly distributed, the marker density corresponded to physical distance from the centromere with a genome average correlation (R^2^) of 0.64 (Table 1). Comparison of the distribution of SSRs on different chromosomes suggested that there were drastic differences among chromosomes. Some chromosomes had much lower correlations such as Gm13 and Gm14 where the R^2^ value was 0.35. The highest correlation (R^2^ = 0.85) was observed for Gm06 (Table 1). Although marker density was very low in the centromeric or pericentromeric regions, chromosomes Gm01, Gm03, Gm05, Gm15, and Gm17 all have at least one marker very near or in the predicted centromeric region (Figure 1).

To understand if the distribution of SSR markers on chromosomes represents a true picture of distribution of genes, we used the physical locations of 46,430 predicted soybean genes from soybean sequence database (www.phytozome.net). A comparison of the percent markers in each section of the chromosomes to the percent genes in each section showed a high average correlation (R^2^ = 0.83) between marker and gene distribution (Figure 2, Table 1). Chromosomes Gm10, Gm14 and Gm20 all had correlations above 0.9 with the highest R^2^ value being 0.94 for Gm20. The lowest R^2^ value of 0.57 was observed for Gm13 (Table 1).

As expected based on high correlations between the distribution of genes and SSR markers, the genes showed similar patterns of distribution as the markers in the distal and proximal regions (Figure 1). The distal 25% of each chromosome contained an average of 50.2% of the genes while the proximal 25% region of each chromosome contained an average of 6.7% of the genes (Table 1). The distal ends ranged from 31.4% of the genes on chromosome Gm13 to 63.6% of the genes on Gm01 (Table 1). The proximal regions ranged from 3.5% of the genes on chromosome Gm06 to 13.4% of the genes on Gm13. In addition, the correlation of gene density with distance from the centromere (R^2^ = 0.83) was higher than the correlation between marker density with distance from the centromere (R^2^ = 0.64) (Table 1).

### Physical distribution of recombination on soybean chromosomes

Comparison of sequence based physical maps and integrated genetic linkage maps using 2188 common markers provided a detailed and precise estimate of recombination at a sub-regional level (Figure 1). In general, recombination was higher in distal regions of the chromosomes (Figure 1, Table 2). Due to lack of recombination in the proximal regions, a large number of markers appeared to be clustered in the centromeric regions of the linkage maps. Although most centromeric and pericentromeric regions were mostly devoid of recombination, a few chromosomes had spikes of very high recombination in the proximal regions (Figure 1, Table 2). Besides the centromeric regions, there were a number of other locations throughout the chromosomes where no or very little recombination occurred (Figure 1). For example, Gm01, Gm14, and Gm18 all showed low recombination toward the middle of the long chromosome arm. Crossing over frequency (CF) was used as a tool to compare distribution of recombination on soybean chromosomes. Among the 20 soybean chromosomes, Gm16 had the lowest CF of 3.3 cM/MB and chromosome Gm07 had the highest CF of 14.8 cM/MB, a greater than 4-fold difference (Table 2). The genome average for CF was 7.2 cM/MB. Chromosome Gm10 had the highest CF of 10.0 cM/MB in the distal 25% and Gm08 had the highest CF of 3.08 cM/MB in the proximal 25% (Table 2). Comparison of the average CF in the distal and proximal regions clearly showed higher recombination in the distal 25% with the average CF value of 8.1 cM/MB compared to 0.4 cM/MB in the proximal 25%. However, the correlation of CF with distance from the centromere was relatively weak with an R^2^ value of 0.21. The highest correlation value of 0.40 was observed for chromosome Gm04. Chromosomes Gm01, Gm07, Gm08, Gm13 and Gm20 showed correlation values below 0.15 (Table 2). The lowest correlation was observed for chromosome Gm20. The number of recombination hotspots, defined as having a CF in excess of 2-fold as compared to the genome average, ranged from 12 on Gm11 to 1 on Gm16, with an average of 6 per chromosome (Table 2, Figure 1).

**Table 2:** Distribution of recombination on soybean chromosomes.

Chromosome #	MLG	Average CF (cM/MB)	Number of recombination Hot Spots*	CF in Distal 25% (cM/Mb)	CF in Proximal 25% (cM/MB)	Correlation of CF with Distance from Centromere
Gm01	D1a	6.9	11	8.2	0.22	0.14
Gm02	D1b	5.7	6	8.3	0.17	0.24
Gm03	N	6.0	5	6.5	0.24	0.25
Gm04	C1	6.3	6	9.7	0.16	0.40
Gm05	A1	6.1	2	8.8	0.08	0.37
Gm06	C2	7.4	11	8.9	0.09	0.23
Gm07	M	14.8	8	9.5	0.04	0.05
Gm08	A2	8.9	6	7.1	3.08	0.06
Gm09	K	4.8	5	6.4	0.21	0.29
Gm10	O	6.9	8	10.0	0.14	0.31
Gm11	B1	10.4	12	9.4	1.16	0.23
Gm12	H	7.0	6	8.8	0.00	0.20
Gm13	F	7.7	4	7.0	0.58	0.12
Gm14	B2	6.4	4	7.9	0.15	0.15
Gm15	E	5.2	4	5.6	0.19	0.33
Gm16	J	3.3	1	6.5	0.05	0.29
Gm17	D2	7.4	9	6.9	0.13	0.20
Gm18	G	8.5	7	9.5	0.55	0.24
Gm19	L	7.0	4	8.1	0.04	0.24
Gm20	I	6.4	4	8.5	0.81	−0.11
Genome Average		7.2	6	8.1	0.40	0.21

CF, Crossing-over Frequency.

*hot spot where crossover frequency (CF) >14.

## Discussion

We used a total of 2188 SSR markers to examine the relationship between the physical distribution of genes, markers and recombination. The distribution of SSR markers closely matched with the distribution of genes. The average correlation between marker and gene distribution was very high with a R^2^ value of 0.83, suggesting a strong relationship between SSRs and gene containing regions (Figure 2, Table 1). SSR markers have been known to be closely associated with gene-rich regions of plant genomes [16]. This relationship between genes and SSR markers has been previously reported in soybean [17]. Findings of this investigation in conjunction with previous data present strong support for the use of SSR markers for gene mapping and cloning.

Physical distribution of SSR markers and putative genes suggest that more markers and genes were present near the telomeres than centromeres, with close to 50% in the distal 25% of the chromosomes and less than 8% in the proximal 25% of the chromosomes (Figure 1, Table 1). Information generated based on the soybean genome sequence also revealed that about 57% of the soybean sequence is repeat rich, a large portion of which is present near the centromeres [28]. Gene containing regions in soybean are reported to be clustered in ∼25% of the genome [30]. Though wheat chromosomes have much more distinct gene clusters, they show a similar pattern of gene distribution in the distal regions with all of the major gene rich regions present in the distal 35% of the chromosomes [19]. Similar marker/gene distributions have been seen in a number of higher plants [18], [20], [21], [22]. Although proximal regions of the chromosomes were low in marker and gene density, a few markers were present in or near the predicted centromeric regions on chromosomes Gm01, Gm03, Gm05, Gm15 and Gm17 (Figure 1, Table 1). However, the presence of genes near the centromere is not improbable, as actively transcribing genes have been discovered in the centromeric regions of both wheat and rice [31], [32]. In wheat approximately 6% of the genes were found in gene poor regions, with some in highly heterochromatic regions [19]. The soybean genome sequence suggests that about one fifth of the predicted genes are present in the repeat rich regions [28]. The presence of markers and genes in or near the centromeres suggests that mapping and cloning genes in these regions is still possible, though more difficult, due to the lower density of markers.

Recombination is a vital component for crop breeding and genetics, as it results in new combinations of genes available for crop improvement. Understanding the distribution of recombination on chromosomes can be a powerful tool to characterize and clone genes. We integrated SSR-based genetic linkage maps to make a comprehensive linkage map. By comparing the linkage map with the sequence based physical map we identified the physical locations of the SSR markers on soybean chromosomes and studied physical distribution of recombination on chromosomes. Generally, high recombination was observed in the telomeric regions as compared to the centromeric regions (Figure 1). The average recombination of the distal 25% of the chromosomes was 200-fold higher than the average recombination of the proximal 25% of the chromosomes (Table 2). These observations are in line with several previous studies, which showed suppressed recombination in centromeric regions [23], [24], [25], [33]. In maize, the recombination rate was highest close to the telomeric ends of chromosomes and was highly suppressed around the centromeres [24], [34]. Recombination varied greatly over the length of the soybean chromosomes, with a weak correlation with the distance from the centromere (R^2^ = 0.21). Chromosomes Gm01, Gm07, Gm08, Gm13 and Gm20 showed no significant correlation between recombination and distance from the centromere (Figure 1, Table 2). Larger genomes like wheat and barley are known to show a higher correlation between rate of recombination and the distance from the centromere [35], [36]. In wheat, recombination was seen to increase at an exponential rate relative to distance from the centromere [26]. In the model plant Arabidopsis, however, very little association was reported between recombination rates and distance from the centromere [37]. The soybean genome is about 8 times bigger than Arabidopsis but about 14 times smaller than wheat [38]. Differences in the distribution of recombination and gene distribution between soybean and large genome species like maize, barley, and wheat may be attributed to differences in genome sizes [25].

In soybean, although the distance from the centromere did not correlate highly with the recombination distribution, hot and cold spots of recombination were observed on all the chromosomes. Hotspots of recombination are expected based on studies of soybean and other genomes where large areas of genetic length occupy small areas of chromosome length, indicating uneven recombination distribution [28], [39]. Distal regions of the chromosomes showed a high rate of recombination as compared to the proximal regions; still the correlation between the distance from centromere and rate of recombination is weak, probably due to unevenness generated by recombination hot and cold spots. Even the distal regions of chromosomes contain large chromosomal regions with barely any recombination.

The physical distribution of marker/genes and recombination on soybean chromosomes showed close association. The chromosomal regions that were high in marker/gene density were also high in recombination. Similar findings have been reported in previous soybean studies, where it was shown that 93% of the recombination occurred in the gene-containing euchromatic regions that account for 43% of the genome [28], [39]. Similar results were also reported in corn, where distribution of genes and recombination showed high correlation; however, not all nonrandom distribution of recombination was explained by gene density [40].

Comparison of genetic and physical maps will enable the development of a clear picture of how physical and genetic distances relate and assist in making decisions about cloning approaches. Recently, approximately 33,000 new putative SSR markers have been developed in soybean [15]. Physical and genetic mapping of these markers in targeted studies will help in narrowing down genes of interest. Although the soybean genome has been sequenced, SSR markers will still play an important role in anchoring genetic maps with a limited use of technology.

## Supporting Information

Figure S1 Comparison of integrated genetic linkage maps with sequence based physical maps for all soybean chromosomes. For creating the integrated linkage maps information was combined from three linkage maps [11], [13], [14]. For generating physical maps soybean sequence information was used (http://www.phytozome.net/soybean). Information about generation of integrated genetic maps and physical maps is presented in Materials/Methods section. The “MapChart” program was used to create maps of each chromosome [29].
(PDF)
