# Supplementary material for: Using Microsatellites to Understand the Physical Distribution of Recombination on Soybean Chromosomes
Source: PLoS One. 2011 Jul 20;6(7):e22306. doi: 10.1371/journal.pone.0022306 (PMC3140510; doi:10.1371/journal.pone.0022306)
Supplement: Figure S1 — Comparison of integrated genetic linkage maps with sequence based physical maps for all soybean chromosomes. For creating the integrated linkage maps information was combined from three linkage maps [11], [13], [14]. For generating physical maps soybean sequence information was used (http://www.phytozome.net/soybean). Information about generation of integrated genetic maps and physical maps is presented in Materials/Methods section. The “MapChart” program was used to create maps of each chromosome [29]. (PDF) [file pone.0022306.s001.pdf]

Figure S1. Comparison of integrated genetic linkage maps with sequence based physical maps for all soybean chromosomes. For creating the integrated linkage maps information was combined from three linkage maps [11,13,14]. For generating physical maps soybean sequence information was used (http://www.phytozome.net/soybean). Information about generation of integrated genetic maps and physical maps is presented in Materials/ Methods section. The “MapChart” program was used to create maps of each chromosome [29].

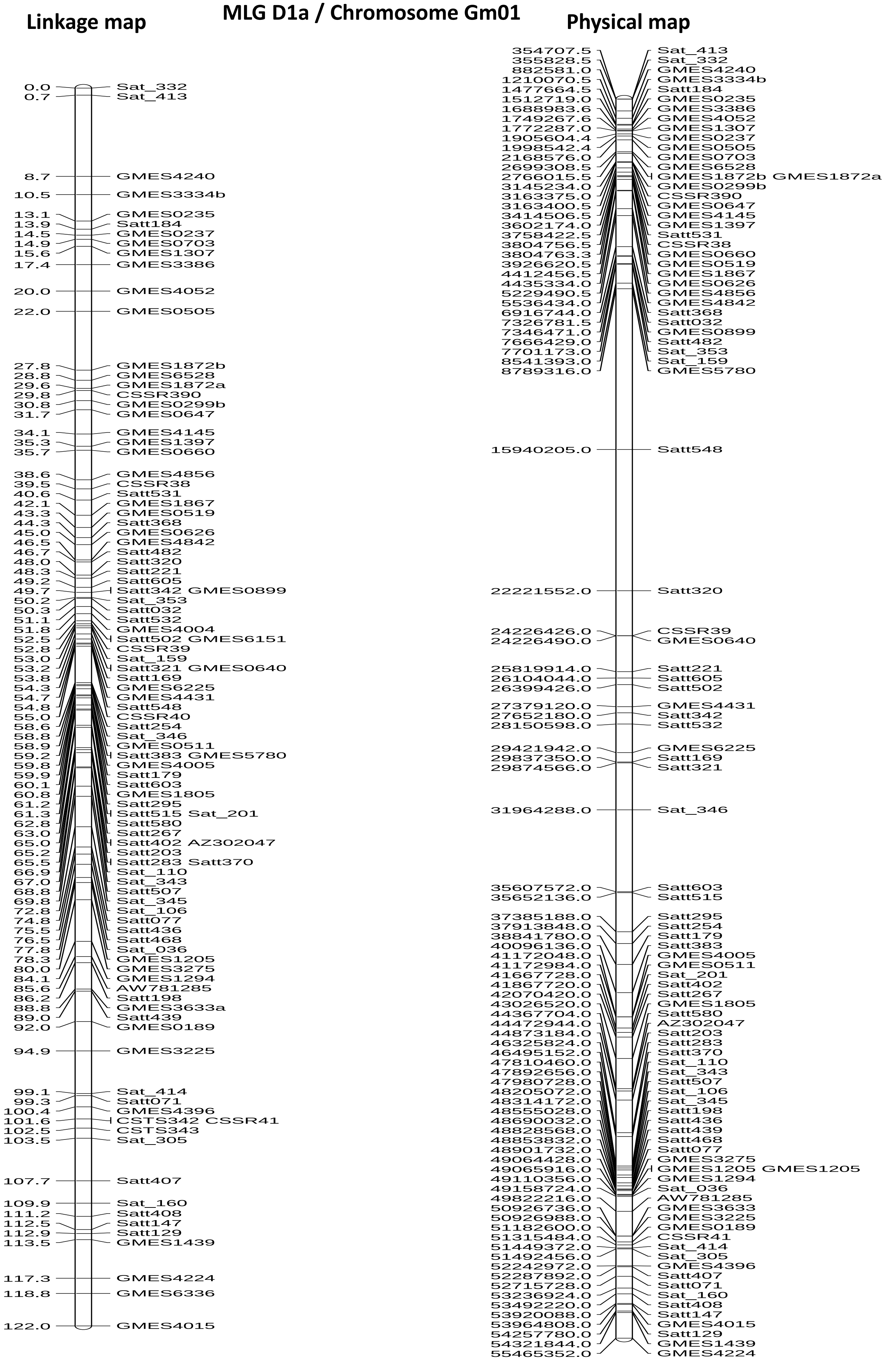
MLG D1a / Chromosome Gm01
Linkage map
0.0 Sat_332
0.7 Sat_413
8.7 GMES4240
10.5 GMES3334b
13.1 GMES0235
13.9 Satt184
14.5 GMES0237
14.9 GMES0703
15.6 GMES1307
17.4 GMES3386
20.0 GMES4052
22.0 GMES0505
27.8 GMES1872b
28.8 GMES6528
29.6 GMES1872a
29.8 CSSR390
30.8 GMES0299b
31.7 GMES0647
34.1 GMES4145
35.3 GMES1397
35.7 GMES0660
38.6 GMES4856
39.5 CSSR38
40.6 Satt531
42.1 GMES1867
43.3 GMES0519
44.3 Satt368
45.0 GMES0626
46.5 GMES4842
46.7 Satt482
48.0 Satt320
48.3 Satt221
49.2 Satt605
49.7 Satt342 GMES0899
50.2 Sat_353
50.3 Satt032
51.1 Satt532
51.8 GMES4004
52.5 Satt502 GMES6151
52.8 CSSR39
53.0 Sat_159
53.2 Satt321 GMES0640
53.8 Satt169
54.3 GMES6225
54.7 GMES4431
54.8 Satt548
55.0 CSSR40
58.6 Satt254
58.8 Sat_346
58.9 GMES0511
59.2 Satt383 GMES5780
59.8 GMES4005
59.9 Satt179
60.1 Satt603
60.8 GMES1805
61.2 Satt295
61.3 Satt515 Sat_201
62.8 Satt580
63.0 Satt267
65.0 Satt402 AZ302047
65.2 Satt203
65.5 Satt283 Satt370
66.9 Sat_110
67.0 Sat_343
68.8 Satt507
69.8 Sat_345
72.8 Sat_106
74.8 Satt077
75.5 Satt436
76.5 Satt468
77.8 Sat_036
78.3 GMES1205
80.0 GMES3275
84.1 GMES1294
85.6 AW781285
86.2 Satt198
88.8 GMES3633a
89.0 Satt439
92.0 GMES0189
94.9 GMES3225
99.1 Sat_414
99.3 Satt071
100.4 GMES4396
101.6 CSTS342 CSSR41
102.5 CSTS343
103.5 Sat_305
107.7 Satt407
109.9 Sat_160
111.2 Satt408
112.5 Satt147
112.9 Satt129
113.5 GMES1439
117.3 GMES4224
118.8 GMES6336
122.0 GMES4015
Physical map
354707.5 Sat_413
355828.5 Sat_332
882581.0 GMES4240
1210070.5 GMES3334b
1477664.5 Satt184
1512719.0 GMES0235
1688983.6 GMES3386
1749267.6 GMES4052
1772287.0 GMES1307
1905604.4 GMES0237
1998542.4 GMES0505
2168576.0 GMES0703
2699308.5 GMES6528
2766015.5 GMES1872b GMES1872a
3145234.0 GMES0299b
3163375.0 CSSR390
3163400.5 GMES0647
3414506.5 GMES4145
3602174.0 GMES1397
3758422.5 Satt531
3804756.5 CSSR38
3804763.3 GMES0660
3926620.5 GMES0519
4412456.5 GMES1867
4435334.0 GMES0626
5229490.5 GMES4856
5536434.0 GMES4842
6916744.0 Satt368
7326781.5 Satt032
7346471.0 GMES0899
7666429.0 Satt482
7701173.0 Sat_353
8541393.0 Sat_159
8789316.0 GMES5780
15940205.0 Satt548
22221552.0 Satt320
24226426.0 CSSR39
24226490.0 GMES0640
25819914.0 Satt221
26104044.0 Satt605
26399426.0 Satt502
27379120.0 GMES4431
27652180.0 Satt342
28150598.0 Satt532
29421942.0 GMES6225
29837350.0 Satt169
29874566.0 Satt321
31964288.0 Sat_346
35607572.0 Satt603
35652136.0 Satt515
37385188.0 Satt295
37913848.0 Satt254
38841780.0 Satt179
40096136.0 Satt383
41172048.0 GMES4005
41172984.0 GMES0511
41667728.0 Sat_201
41867720.0 Satt402
42070420.0 Satt267
43026520.0 GMES1805
44367704.0 Satt580
44472944.0 AZ302047
44873184.0 Satt203
46325824.0 Satt283
46495152.0 Satt370
47810460.0 Sat_110
47892656.0 Sat_343
47980728.0 Satt507
48205072.0 Sat_106
48314172.0 Sat_345
48555028.0 Satt198
48690032.0 Satt436
48828568.0 Satt439
48853832.0 Satt468
48901732.0 Satt077
49064428.0 GMES3275
49065916.0 GMES1205 GMES1205
49110356.0 GMES1294
49158724.0 Sat_036
49822216.0 AW781285
50926736.0 GMES3633
50926988.0 GMES3225
51182600.0 GMES0189
51315484.0 CSSR41
51449372.0 Sat_414
51492456.0 Sat_305
52242972.0 GMES4396
52287892.0 Satt407
52715728.0 Satt071
53236924.0 Sat_160
53492220.0 Satt408
53920088.0 Satt147
53964808.0 GMES4015
54257780.0 Satt129
54321844.0 GMES1439
55465352.0 GMES4224

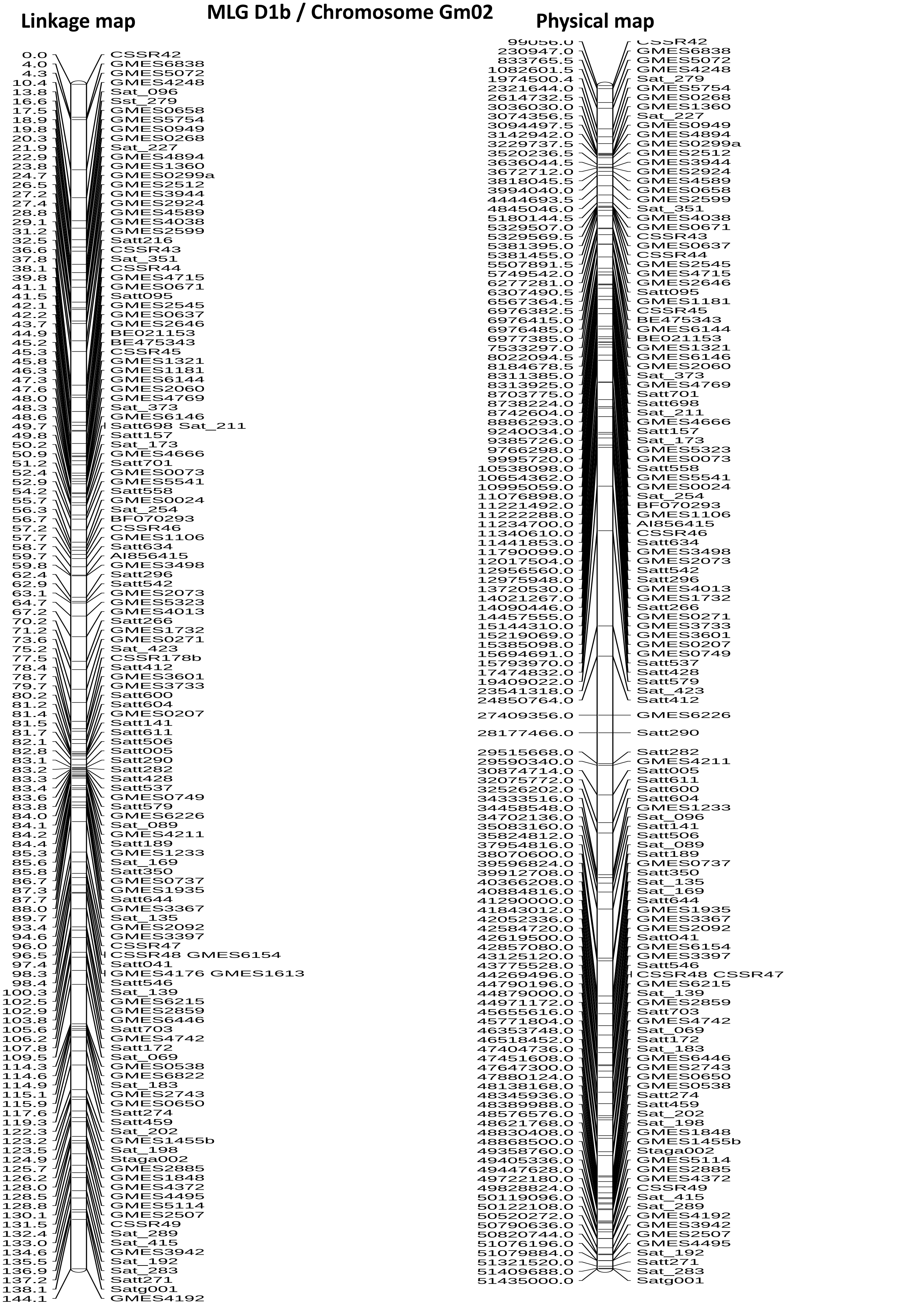
MLG D1b / Chromosome Gm02
Linkage map
0.0 CSSR42
4.0 GMES6838
4.3 GMES5072
10.4 GMES4248
13.8 Sat_096
16.6 Sst_279
17.5 GMES0658
18.9 GMES5754
19.8 GMES0949
20.3 GMES0268
21.9 Sat_227
22.9 GMES4894
23.8 GMES1360
24.7 GMES0299a
26.5 GMES2512
27.2 GMES3944
27.4 GMES2924
28.8 GMES4589
29.1 GMES4038
31.2 GMES2599
32.5 Satt216
36.6 CSSR43
37.8 Sat_351
38.1 CSSR44
39.8 GMES4715
41.1 GMES0671
41.5 Satt095
42.1 GMES2545
42.2 GMES0637
43.7 GMES2646
44.9 BE021153
45.2 BE475343
45.3 CSSR45
45.8 GMES1321
46.3 GMES1181
47.3 GMES6144
47.6 GMES2060
48.0 GMES4769
48.3 Sat_373
48.6 GMES6146
49.7 Satt698 Sat_211
49.8 Satt157
50.2 Sat_173
50.9 GMES4666
51.2 Satt701
52.4 GMES0073
52.9 GMES5541
54.2 Satt558
55.7 GMES0024
56.3 Sat_254
56.7 BF070293
57.2 CSSR46
57.7 GMES1106
58.7 Satt634
59.7 AI856415
59.8 GMES3498
62.4 Satt296
62.9 Satt542
63.1 GMES2073
64.7 GMES5323
67.2 GMES4013
70.2 Satt266
71.2 GMES1732
73.6 GMES0271
75.2 Sat_423
77.5 CSSR178b
78.4 Satt412
78.7 GMES3601
79.7 GMES3733
80.2 Satt600
81.2 Satt604
81.4 GMES0207
81.5 Satt141
81.7 Satt611
82.1 Satt506
82.8 Satt005
83.1 Satt290
83.2 Satt282
83.3 Satt428
83.4 Satt537
83.6 GMES0749
83.8 Satt579
84.0 GMES6226
84.1 Sat_089
84.2 GMES4211
84.4 Satt189
85.3 GMES1233
85.6 Sat_169
85.8 Satt350
86.7 GMES0737
87.3 GMES1935
87.7 Satt644
88.0 GMES3367
89.7 Sat_135
93.4 GMES2092
94.6 GMES3397
96.0 CSSR47
96.5 CSSR48 GMES6154
97.4 Satt041
98.3 GMES4176 GMES1613
98.4 Satt546
100.3 Sat_139
102.5 GMES6215
102.9 GMES2859
103.8 GMES6446
105.6 Satt703
106.2 GMES4742
107.8 Satt172
109.5 Sat_069
114.3 GMES0538
114.6 GMES6822
114.9 Sat_183
115.1 GMES2743
115.9 GMES0650
117.6 Satt274
119.3 Satt459
122.3 Sat_202
123.2 GMES1455b
123.5 Sat_198
124.9 Staga002
125.7 GMES2885
126.2 GMES1848
128.0 GMES4372
128.5 GMES4495
128.8 GMES5114
130.1 GMES2507
131.5 CSSR49
132.4 Sat_289
133.0 Sat_415
134.6 GMES3942
135.5 Sat_192
136.9 Sat_283
137.2 Satt271
138.1 Satg001
144.1 GMES4192
Physical map
99056.0 CSSR42
230947.0 GMES6838
833765.5 GMES5072
1082601.5 GMES4248
1974500.4 Sat_279
2321644.0 GMES5754
2614732.5 GMES0268
3036030.0 GMES1360
3074356.5 Sat_227
3094497.5 GMES0949
3142942.0 GMES4894
3229737.5 GMES0299a
3520236.5 GMES2512
3636044.5 GMES3944
3672712.0 GMES2924
3818045.5 GMES4589
3994040.0 GMES0658
4444693.5 GMES2599
4845046.0 Sat_351
5180144.5 GMES4038
5329507.0 GMES0671
5329569.5 CSSR43
5381395.0 GMES0637
5381455.0 CSSR44
5507891.5 GMES2545
5749542.0 GMES4715
6277281.0 GMES2646
6307490.5 Satt095
6567364.5 GMES1181
6976382.5 CSSR45
6976415.0 BE475343
6976485.0 GMES6144
6977385.0 BE021153
7533297.0 GMES1321
8022094.5 GMES6146
8184678.5 GMES2060
8311385.0 Sat_373
8313925.0 GMES4769
8703775.0 Satt701
8738224.0 Satt698
8742604.0 Sat_211
8886293.0 GMES4666
9240034.0 Satt157
9385726.0 Sat_173
9766298.0 GMES5323
9995720.0 GMES0073
10538098.0 Satt558
10654362.0 GMES5541
10995059.0 GMES0024
11076898.0 Sat_254
11221492.0 BF070293
11222288.0 GMES1106
11234700.0 AI856415
11340610.0 CSSR46
11441853.0 Satt634
11790099.0 GMES3498
12017504.0 GMES2073
12956560.0 Satt542
12975948.0 Satt296
13720530.0 GMES4013
14021267.0 GMES1732
14090446.0 Satt266
14457555.0 GMES0271
15144310.0 GMES3733
15219069.0 GMES3601
15385098.0 GMES0207
15694691.0 GMES0749
15793970.0 Satt537
17474832.0 Satt428
19409022.0 Satt579
23541318.0 Sat_423
24850764.0 Satt412
27409356.0 GMES6226
28177466.0 Satt290
29515668.0 Satt282
29590340.0 GMES4211
30874714.0 Satt005
32075772.0 Satt611
32526202.0 Satt600
34333516.0 Satt604
34458548.0 GMES1233
34702136.0 Sat_096
35083160.0 Satt141
35824812.0 Satt506
37954816.0 Sat_089
38070600.0 Satt189
39596824.0 GMES0737
39912708.0 Satt350
40366208.0 Sat_135
40884816.0 Sat_169
41290000.0 Satt644
41843012.0 GMES1935
42052336.0 GMES3367
42584720.0 GMES2092
42619500.0 Satt041
42857080.0 GMES6154
43125120.0 GMES3397
43775528.0 Satt546
44269496.0 CSSR48 CSSR47
44790196.0 GMES6215
44879000.0 Sat_139
44971172.0 GMES2859
45655616.0 Satt703
45771804.0 GMES4742
46353748.0 Sat_069
46518452.0 Satt172
47404736.0 Sat_183
47451608.0 GMES6446
47647300.0 GMES2743
47880124.0 GMES0650
48138168.0 GMES0538
48345936.0 Satt274
48389988.0 Satt459
48576576.0 Sat_202
48621768.0 Sat_198
48830408.0 GMES1848
48868500.0 GMES1455b
49358760.0 Staga002
49405336.0 GMES5114
49447628.0 GMES2885
49722180.0 GMES4372
49828824.0 CSSR49
50119096.0 Sat_415
50122108.0 Sat_289
50520272.0 GMES4192
50790636.0 GMES3942
50820744.0 GMES2507
51076196.0 GMES4495
51079884.0 Sat_192
51321520.0 Satt271
51409688.0 Sat_283
51435000.0 Satg001

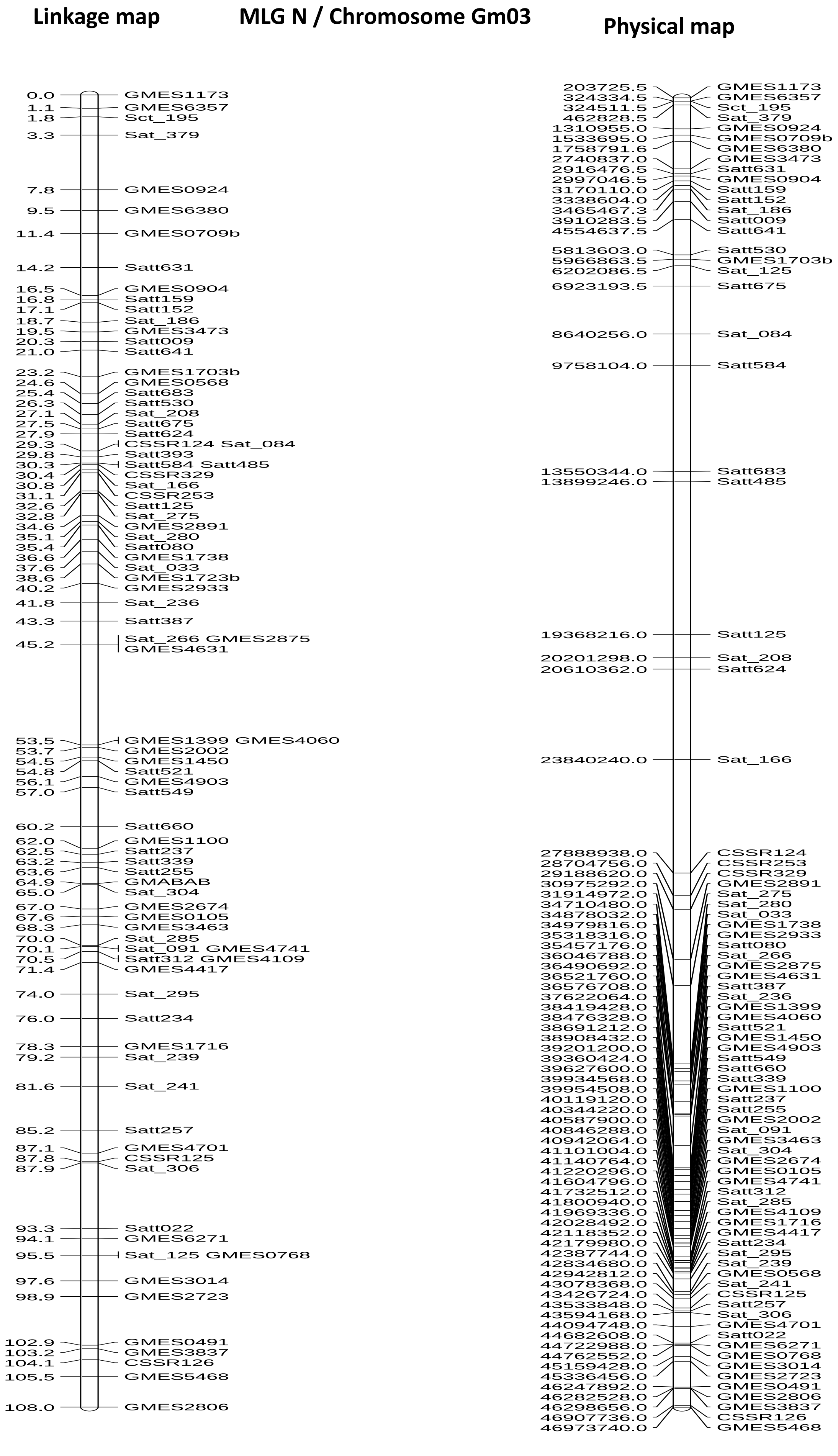

Linkage map
MLG N / Chromosome Gm03
Physical map
0.0 GMES1173
1.1 GMES6357
1.8 Sct_195
3.3 Sat_379
7.8 GMES0924
9.5 GMES6380
11.4 GMES0709b
14.2 Satt631
16.5 GMES0904
16.8 Satt159
17.1 Satt152
18.7 Sat_186
19.5 GMES3473
20.3 Satt009
21.0 Satt641
23.2 GMES1703b
24.6 GMES0568
25.4 Satt683
26.3 Satt530
27.1 Sat_208
27.5 Satt675
27.9 Satt624
29.3 CSSR124 Sat_084
29.8 Satt393
30.3 Satt584 Satt485
30.4 CSSR329
30.8 Sat_166
31.1 CSSR253
32.6 Satt125
32.8 Sat_275
34.6 GMES2891
35.1 Sat_280
35.4 Satt080
36.6 GMES1738
37.6 Sat_033
38.6 GMES1723b
40.2 GMES2933
41.8 Sat_236
43.3 Satt387
45.2 Sat_266 GMES2875
GMES4631
53.5 GMES1399 GMES4060
53.7 GMES2002
54.5 GMES1450
54.8 Satt521
56.1 GMES4903
57.0 Satt549
60.2 Satt660
62.0 GMES1100
62.5 Satt237
63.2 Satt339
63.6 Satt255
64.9 GMABAB
65.0 Sat_304
67.0 GMES2674
67.6 GMES0105
68.3 GMES3463
70.0 Sat_285
70.1 Sat_091 GMES4741
70.5 Satt312 GMES4109
71.4 GMES4417
74.0 Sat_295
76.0 Satt234
78.3 GMES1716
79.2 Sat_239
81.6 Sat_241
85.2 Satt257
87.1 GMES4701
87.8 CSSR125
87.9 Sat_306
93.3 Satt022
94.1 GMES6271
95.5 Sat_125 GMES0768
97.6 GMES3014
98.9 GMES2723
102.9 GMES0491
103.2 GMES3837
104.1 CSSR126
105.5 GMES5468
108.0 GMES2806
203725.5 GMES1173
324334.5 GMES6357
324511.5 Sct_195
462828.5 Sat_379
1310955.0 GMES0924
1533695.0 GMES0709b
1758791.6 GMES6380
2740837.0 GMES3473
2916476.5 Satt631
2997046.5 GMES0904
3170110.0 Satt159
3338604.0 Satt152
3465467.3 Sat_186
3910283.5 Satt009
4554637.5 Satt641
5813603.0 Satt530
5966863.5 GMES1703b
6202086.5 Sat_125
6923193.5 Satt675
8640256.0 Sat_084
9758104.0 Satt584
13550344.0 Satt683
13899246.0 Satt485
19368216.0 Satt125
20201298.0 Sat_208
20610362.0 Satt624
23840240.0 Sat_166
27888938.0 CSSR124
28704756.0 CSSR253
29188620.0 CSSR329
30975292.0 GMES2891
31914972.0 Sat_275
34710480.0 Sat_280
34878032.0 Sat_033
34979816.0 GMES1738
35318316.0 GMES2933
35457176.0 Satt080
36046788.0 Sat_266
36490692.0 GMES2875
36521760.0 GMES4631
36576708.0 Satt387
37622064.0 Sat_236
38419428.0 GMES1399
38476328.0 GMES4060
38691212.0 Satt521
38908432.0 GMES1450
39201200.0 GMES4903
39360424.0 Satt549
39627600.0 Satt660
39934568.0 Satt339
39954508.0 GMES1100
40119120.0 Satt237
40344220.0 Satt255
40587900.0 GMES2002
40846288.0 Sat_091
40942064.0 GMES3463
41101004.0 Sat_304
41140764.0 GMES2674
41220296.0 GMES0105
41604796.0 GMES4741
41732512.0 Satt312
41800940.0 Sat_285
41969336.0 GMES4109
42028492.0 GMES1716
42118352.0 GMES4417
42179980.0 Satt234
42387744.0 Sat_295
42834680.0 Sat_239
42942812.0 GMES0568
43078368.0 Sat_241
43426724.0 CSSR125
43533848.0 Satt257
43594168.0 Sat_306
44094748.0 GMES4701
44682608.0 Satt022
44722988.0 GMES6271
44762552.0 GMES0768
45159428.0 GMES3014
45336456.0 GMES2723
46247892.0 GMES0491
46282528.0 GMES2806
46298656.0 GMES3837
46907736.0 CSSR126
46973740.0 GMES5468

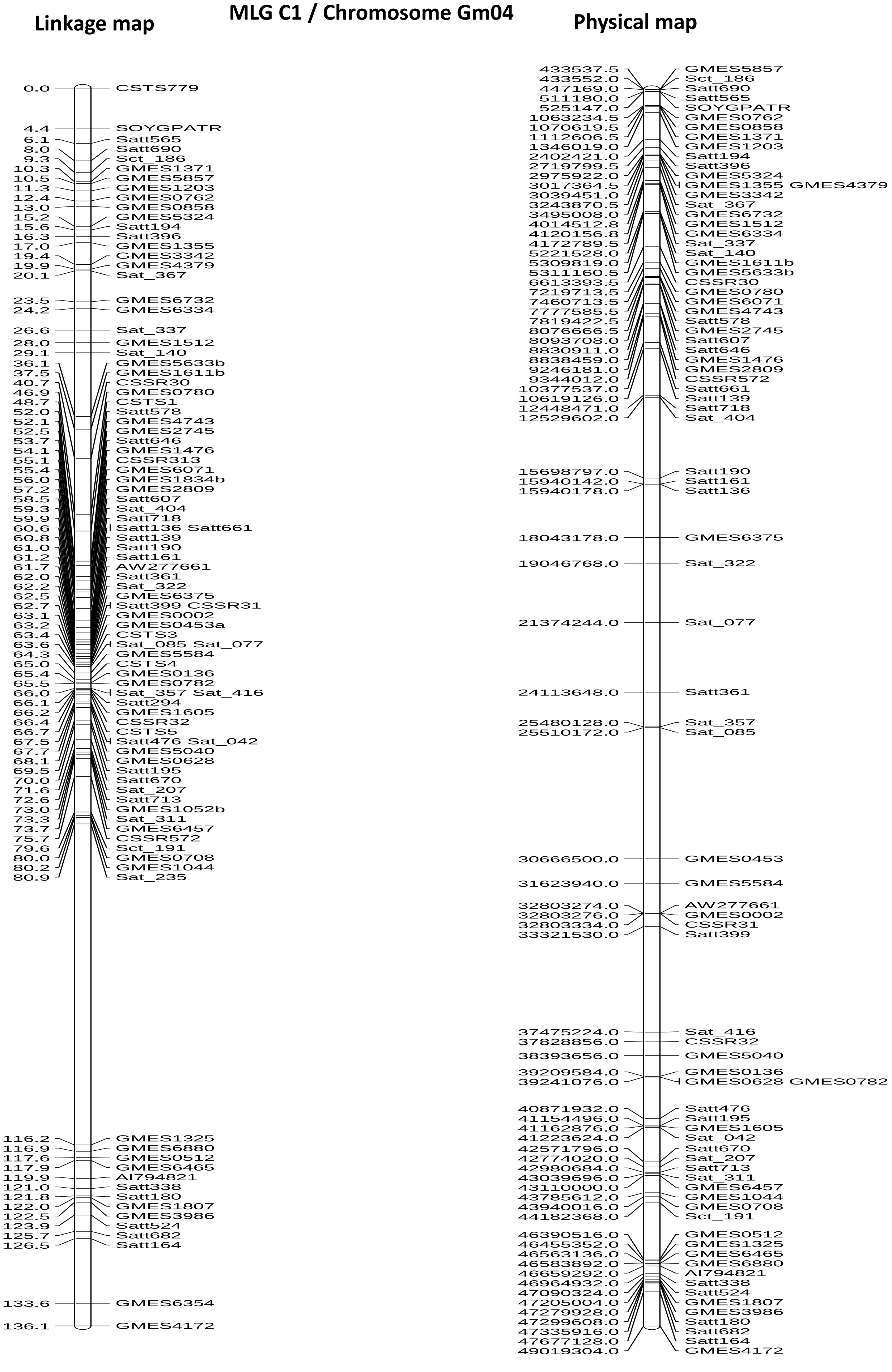
MLG C1 / Chromosome Gm04
Linkage map
0.0 CSTS779
4.4 SOYGPATR
6.1 Satt565
8.0 Satt690
9.3 Sct_186
10.3 GMES1371
10.5 GMES5857
11.3 GMES1203
12.4 GMES0762
13.0 GMES0858
15.2 GMES5324
15.6 Satt194
16.3 Satt396
17.0 GMES1355
19.4 GMES3342
19.9 GMES4379
20.1 Sat_367
23.5 GMES6732
24.2 GMES6334
26.6 Sat_337
28.0 GMES1512
29.1 Sat_140
36.1 GMES5633b
37.5 GMES1611b
40.7 CSSR30
46.9 GMES0780
48.7 CSTS1
52.0 Satt578
52.1 GMES4743
52.5 GMES2745
53.7 Satt646
54.1 GMES1476
55.1 CSSR313
55.4 GMES6071
56.0 GMES1834b
57.2 GMES2809
58.5 Satt607
59.3 Sat_404
59.9 Satt718
60.6 Satt136 Satt661
60.8 Satt139
61.0 Satt190
61.2 Satt161
61.7 AW277661
62.0 Satt361
62.2 Sat_322
62.5 GMES6375
62.7 Satt399 CSSR31
63.1 GMES0002
63.2 GMES0453a
63.4 CSTS3
63.6 Sat_085 Sat_077
64.3 GMES5584
65.0 CSTS4
65.4 GMES0136
65.5 GMES0782
66.0 Sat_357 Sat_416
66.1 Satt294
66.2 GMES1605
66.4 CSSR32
66.7 CSTS5
67.5 Satt476 Sat_042
67.7 GMES5040
68.1 GMES0628
69.5 Satt195
70.0 Satt670
71.6 Sat_207
72.6 Satt713
73.0 GMES1052b
73.3 Sat_311
73.7 GMES6457
75.7 CSSR572
79.6 Sct_191
80.0 GMES0708
80.2 GMES1044
80.9 Sat_235
116.2 GMES1325
116.9 GMES6880
117.6 GMES0512
117.9 GMES6465
119.9 AI794821
121.0 Satt338
121.8 Satt180
122.0 GMES1807
122.5 GMES3986
123.9 Satt524
125.7 Satt682
126.5 Satt164
133.6 GMES6354
136.1 GMES4172
Physical map
433537.5 GMES5857
433552.0 Sct_186
447169.0 Satt690
511180.0 Satt565
525147.0 SOYGPATR
1063234.5 GMES0762
1070619.5 GMES0858
1112606.5 GMES1371
1346019.0 GMES1203
2402421.0 Satt194
2719799.5 Satt396
2975922.0 GMES5324
3017364.5 GMES1355 GMES4379
3039451.0 GMES3342
3243870.5 Sat_367
3495008.0 GMES6732
4014512.8 GMES1512
4120156.8 GMES6334
4172789.5 Sat_337
5221528.0 Sat_140
5309819.0 GMES1611b
5311160.5 GMES5633b
6613393.5 CSSR30
7219713.5 GMES0780
7460713.5 GMES6071
7777585.5 GMES4743
7819422.5 Satt578
8076666.5 GMES2745
8093708.0 Satt607
8830911.0 Satt646
8838459.0 GMES1476
9246181.0 GMES2809
9344012.0 CSSR572
10377537.0 Satt661
10619126.0 Satt139
12448471.0 Satt718
12529602.0 Sat_404
15698797.0 Satt190
15940142.0 Satt161
15940178.0 Satt136
18043178.0 GMES6375
19046768.0 Sat_322
21374244.0 Sat_077
24113648.0 Satt361
25480128.0 Sat_357
25510172.0 Sat_085
30666500.0 GMES0453
31623940.0 GMES5584
32803274.0 AW277661
32803276.0 GMES0002
32803334.0 CSSR31
33321530.0 Satt399
37475224.0 Sat_416
37828856.0 CSSR32
38393656.0 GMES5040
39209584.0 GMES0136
39241076.0 GMES0628 GMES0782
40871932.0 Satt476
41154496.0 Satt195
41162876.0 GMES1605
41223624.0 Sat_042
42571796.0 Satt670
42774020.0 Sat_207
42980684.0 Satt713
43039696.0 Sat_311
43110000.0 GMES6457
43785612.0 GMES1044
43940016.0 GMES0708
44182368.0 Sct_191
46390516.0 GMES0512
46455352.0 GMES1325
46563136.0 GMES6465
46583892.0 GMES6880
46659292.0 AI794821
46964932.0 Satt338
47090324.0 Satt524
47205004.0 GMES1807
47279928.0 GMES3986
47299608.0 Satt180
47335916.0 Satt682
47677128.0 Satt164
49019304.0 GMES4172

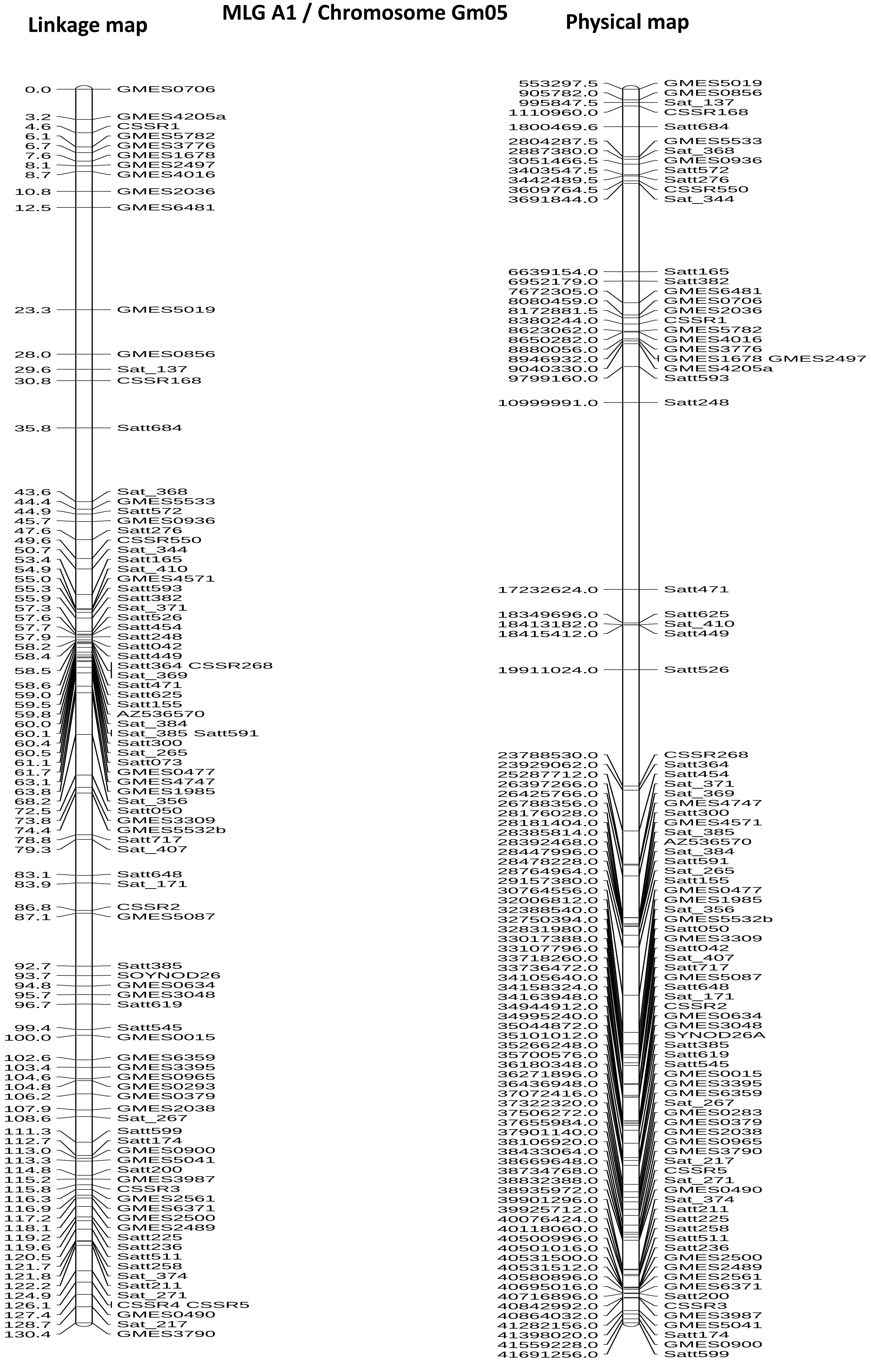
MLG A1 / Chromosome Gm05
Linkage map
0.0 GMES0706
3.2 GMES4205a
4.6 CSSR1
6.1 GMES5782
6.7 GMES3776
7.6 GMES1678
8.1 GMES2497
8.7 GMES4016
10.8 GMES2036
12.5 GMES6481
23.3 GMES5019
28.0 GMES0856
29.6 Sat_137
30.8 CSSR168
35.8 Satt684
43.6 Sat_368
44.4 GMES5533
44.9 Satt572
45.7 GMES0936
47.6 Satt276
49.6 CSSR550
50.7 Sat_344
53.4 Satt165
54.9 Sat_410
55.0 GMES4571
55.3 Satt593
55.9 Satt382
57.3 Sat_371
57.6 Satt526
57.7 Satt454
57.9 Satt248
58.2 Satt042
58.4 Satt449
58.5 Satt364 CSSR268
Sat_369
58.6 Satt471
59.0 Satt625
59.5 Satt155
59.8 AZ536570
60.0 Sat_384
60.1 Sat_385 Satt591
60.4 Satt300
60.5 Sat_265
61.1 Satt073
61.7 GMES0477
63.1 GMES4747
63.8 GMES1985
68.2 Sat_356
72.5 Satt050
73.8 GMES3309
74.4 GMES5532b
78.8 Satt717
79.3 Sat_407
83.1 Satt648
83.9 Sat_171
86.8 CSSR2
87.1 GMES5087
92.7 Satt385
93.7 SOYNOD26
94.8 GMES0634
95.7 GMES3048
96.7 Satt619
99.4 Satt545
100.0 GMES0015
102.6 GMES6359
103.4 GMES3395
104.6 GMES0965
104.8 GMES0293
106.2 GMES0379
107.9 GMES2038
108.6 Sat_267
111.3 Satt599
112.7 Satt174
113.0 GMES0900
113.3 GMES5041
114.8 Satt200
115.2 GMES3987
115.8 CSSR3
116.3 GMES2561
116.9 GMES6371
117.2 GMES2500
118.1 GMES2489
119.2 Satt225
119.6 Satt236
120.5 Satt511
121.7 Satt258
121.8 Sat_374
122.2 Satt211
124.9 Sat_271
126.1 CSSR4 CSSR5
127.4 GMES0490
128.7 Sat_217
130.4 GMES3790
Physical map
553297.5 GMES5019
905782.0 GMES0856
995847.5 Sat_137
1110960.0 CSSR168
1800469.6 Satt684
2804287.5 GMES5533
2887380.0 Sat_368
3051466.5 GMES0936
3403547.5 Satt572
3442489.5 Satt276
3609764.5 CSSR550
3691844.0 Sat_344
6639154.0 Satt165
6952179.0 Satt382
7672305.0 GMES6481
8080459.0 GMES0706
8172881.5 GMES2036
8380244.0 CSSR1
8623062.0 GMES5782
8650282.0 GMES4016
8880056.0 GMES3776
8946932.0 GMES1678 GMES2497
9040330.0 GMES4205a
9799160.0 Satt593
10999991.0 Satt248
17232624.0 Satt471
18349696.0 Satt625
18413182.0 Sat_410
18415412.0 Satt449
19911024.0 Satt526
23788530.0 CSSR268
23929062.0 Satt364
25287712.0 Satt454
26397266.0 Sat_371
26425766.0 Sat_369
26788356.0 GMES4747
28176028.0 Satt300
28181404.0 GMES4571
28385814.0 Sat_385
28392468.0 AZ536570
28447996.0 Sat_384
28478228.0 Satt591
28764964.0 Sat_265
29157380.0 Satt155
30764556.0 GMES0477
32006812.0 GMES1985
32388540.0 Sat_356
32750394.0 GMES5532b
32831980.0 Satt050
33017388.0 GMES3309
33107796.0 Satt042
33718260.0 Sat_407
33736472.0 Satt717
34105640.0 GMES5087
34158324.0 Satt648
34163948.0 Sat_171
34944912.0 CSSR2
34995240.0 GMES0634
35044872.0 GMES3048
35101012.0 SYNOD26A
35266248.0 Satt385
35700576.0 Satt619
36180348.0 Satt545
36271896.0 GMES0015
36436948.0 GMES3395
37072416.0 GMES6359
37322320.0 Sat_267
37506272.0 GMES0283
37655984.0 GMES0379
37901140.0 GMES2038
38106920.0 GMES0965
38433064.0 GMES3790
38669648.0 Sat_217
38734768.0 CSSR5
38832388.0 Sat_271
38935972.0 GMES0490
39901296.0 Sat_374
39925712.0 Satt211
40076424.0 Satt225
40118060.0 Satt258
40500996.0 Satt511
40501016.0 Satt236
40531500.0 GMES2500
40531512.0 GMES2489
40580896.0 GMES2561
40695016.0 GMES6371
40716896.0 Satt200
40842992.0 CSSR3
40864032.0 GMES3987
41282156.0 GMES5041
41398020.0 Satt174
41559228.0 GMES0900
41691256.0 Satt599

MLG C2 / Chromosome Gm06

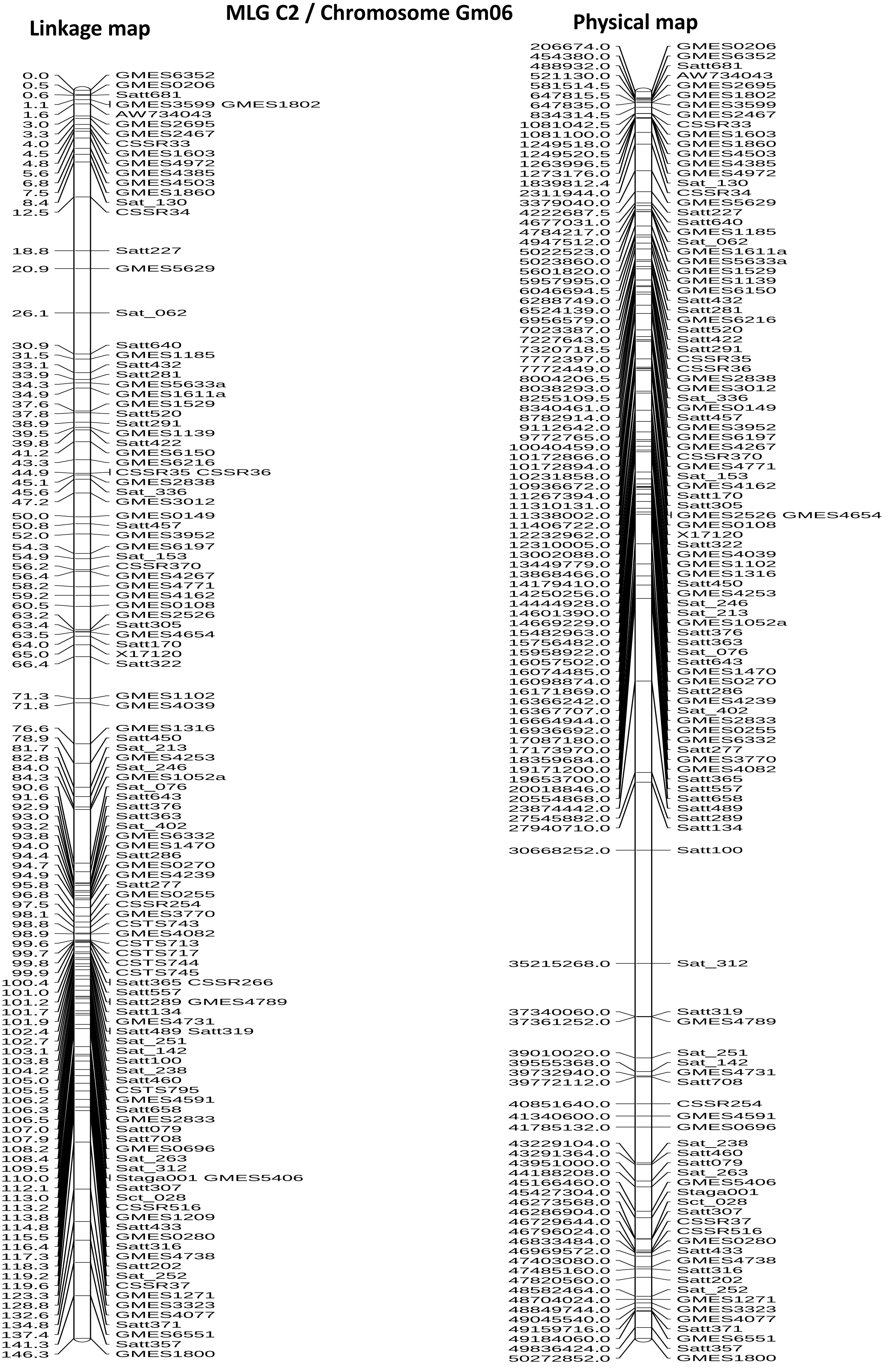

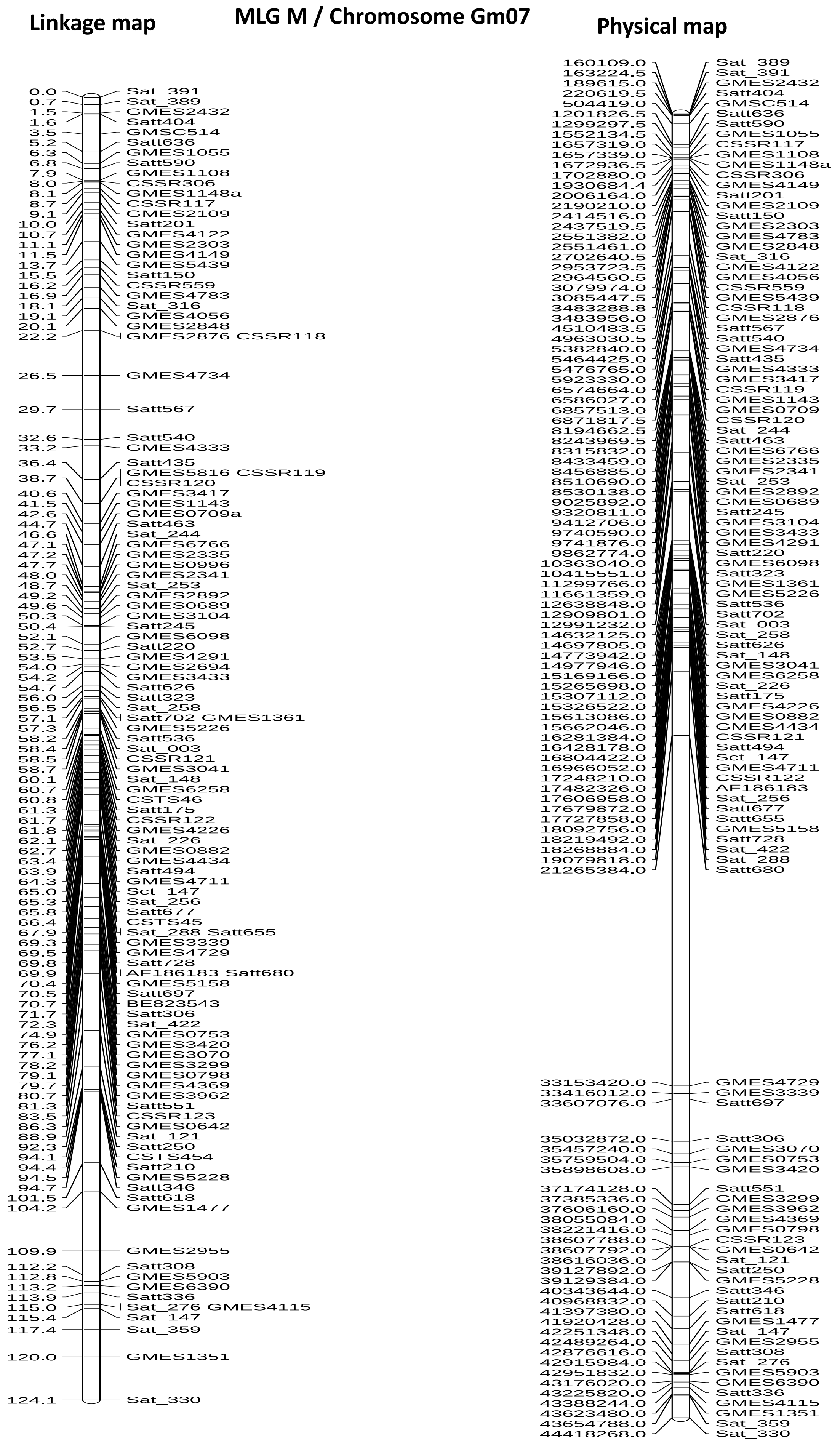
Linkage map
MLG M / Chromosome Gm07
Physical map
0.0 Sat_391
0.7 Sat_389
1.5 GMES2432
1.6 Satt404
3.5 GMSC514
5.2 Satt636
6.3 GMES1055
6.8 Satt590
7.9 GMES1108
8.0 CSSR306
8.1 GMES1148a
8.7 CSSR117
9.1 GMES2109
10.0 Satt201
10.7 GMES4122
11.1 GMES2303
11.5 GMES4149
13.7 GMES5439
15.5 Satt150
16.2 CSSR559
16.9 GMES4783
18.1 Sat_316
19.1 GMES4056
20.1 GMES2848
22.2 GMES2876 CSSR118
26.5 GMES4734
29.7 Satt567
32.6 Satt540
33.2 GMES4333
36.4 Satt435
GMES5816 CSSR119
38.7 CSSR120
40.6 GMES3417
41.5 GMES1143
42.6 GMES0709a
44.7 Satt463
46.6 Sat_244
47.1 GMES6766
47.2 GMES2335
47.7 GMES0996
48.0 GMES2341
48.7 Sat_253
49.2 GMES2892
49.6 GMES0689
50.3 GMES3104
50.4 Satt245
52.1 GMES6098
52.7 Satt220
53.5 GMES4291
54.0 GMES2694
54.2 GMES3433
54.7 Satt626
56.0 Satt323
56.5 Sat_258
57.1 Satt702 GMES1361
57.3 GMES5226
58.2 Satt536
58.4 Sat_003
58.5 CSSR121
58.7 GMES3041
60.1 Sat_148
60.7 GMES6258
60.8 CSTS46
61.3 Satt175
61.7 CSSR122
61.8 GMES4226
62.1 Sat_226
62.7 GMES0882
63.4 GMES4434
63.9 Satt494
64.3 GMES4711
65.0 Sct_147
65.3 Sat_256
65.8 Satt677
66.4 CSTS45
67.9 Sat_288 Satt655
69.3 GMES3339
69.5 GMES4729
69.8 Satt728
69.9 AF186183 Satt680
70.4 GMES5158
70.5 Satt697
70.7 BE823543
71.7 Satt306
72.3 Sat_422
74.9 GMES0753
76.2 GMES3420
77.1 GMES3070
78.2 GMES3299
79.1 GMES0798
79.7 GMES4369
80.7 GMES3962
81.3 Satt551
83.5 CSSR123
86.3 GMES0642
88.9 Sat_121
92.3 Satt250
94.1 CSTS454
94.4 Satt210
94.5 GMES5228
94.7 Satt346
101.5 Satt618
104.2 GMES1477
109.9 GMES2955
112.2 Satt308
112.8 GMES5903
113.2 GMES6390
113.9 Satt336
115.0 Sat_276 GMES4115
115.4 Sat_147
117.4 Sat_359
120.0 GMES1351
124.1 Sat_330
160109.0 Sat_389
163224.5 Sat_391
189615.0 GMES2432
220619.5 Satt404
504419.0 GMSC514
1201826.5 Satt636
1299297.5 Satt590
1552134.5 GMES1055
1657319.0 CSSR117
1657339.0 GMES1108
1672936.5 GMES1148a
1702880.0 CSSR306
1930684.4 GMES4149
2006164.0 Satt201
2190210.0 GMES2109
2414516.0 Satt150
2437519.5 GMES2303
2551382.0 GMES4783
2551461.0 GMES2848
2702640.5 Sat_316
2953723.5 GMES4122
2964560.5 GMES4056
3079974.0 CSSR559
3085447.5 GMES5439
3483288.8 CSSR118
3483956.0 GMES2876
4510483.5 Satt567
4963030.5 Satt540
5382840.0 GMES4734
5464425.0 Satt435
5476765.0 GMES4333
5923330.0 GMES3417
6574664.0 CSSR119
6586027.0 GMES1143
6857513.0 GMES0709
6871817.5 CSSR120
8194662.5 Sat_244
8243969.5 Satt463
8315832.0 GMES6766
8433459.0 GMES2335
8456885.0 GMES2341
8510690.0 Sat_253
8530138.0 GMES2892
9025892.0 GMES0689
9320811.0 Satt245
9412706.0 GMES3104
9740590.0 GMES3433
9741876.0 GMES4291
9862774.0 Satt220
10363040.0 GMES6098
10415551.0 Satt323
11299766.0 GMES1361
11661359.0 GMES5226
12638848.0 Satt536
12909801.0 Satt702
12991232.0 Sat_003
14632125.0 Sat_258
14697805.0 Satt626
14773942.0 Sat_148
14977946.0 GMES3041
15169166.0 GMES6258
15265698.0 Sat_226
15307112.0 Satt175
15326522.0 GMES4226
15613086.0 GMES0882
15662046.0 GMES4434
16281384.0 CSSR121
16428178.0 Satt494
16804422.0 Sct_147
16966052.0 GMES4711
17248210.0 CSSR122
17482326.0 AF186183
17606958.0 Sat_256
17679872.0 Satt677
17727858.0 Satt655
18092756.0 GMES5158
18219492.0 Satt728
18268884.0 Sat_422
19079818.0 Sat_288
21265384.0 Satt680
33153420.0 GMES4729
33416012.0 GMES3339
33607076.0 Satt697
35032872.0 Satt306
35457240.0 GMES3070
35759504.0 GMES0753
35898608.0 GMES3420
37174128.0 Satt551
37385336.0 GMES3299
37606160.0 GMES3962
38055084.0 GMES4369
38221416.0 GMES0798
38607788.0 CSSR123
38607792.0 GMES0642
38616036.0 Sat_121
39127892.0 Satt250
39129384.0 GMES5228
40343644.0 Satt346
40968832.0 Satt210
41397380.0 Satt618
41920428.0 GMES1477
42251348.0 Sat_147
42489264.0 GMES2955
42876616.0 Satt308
42915984.0 Sat_276
42951832.0 GMES5903
43176020.0 GMES6390
43225820.0 Satt336
43388244.0 GMES4115
43623480.0 GMES1351
43654788.0 Sat_359
44418268.0 Sat_330

MLG A2 / Chromosome Gm08

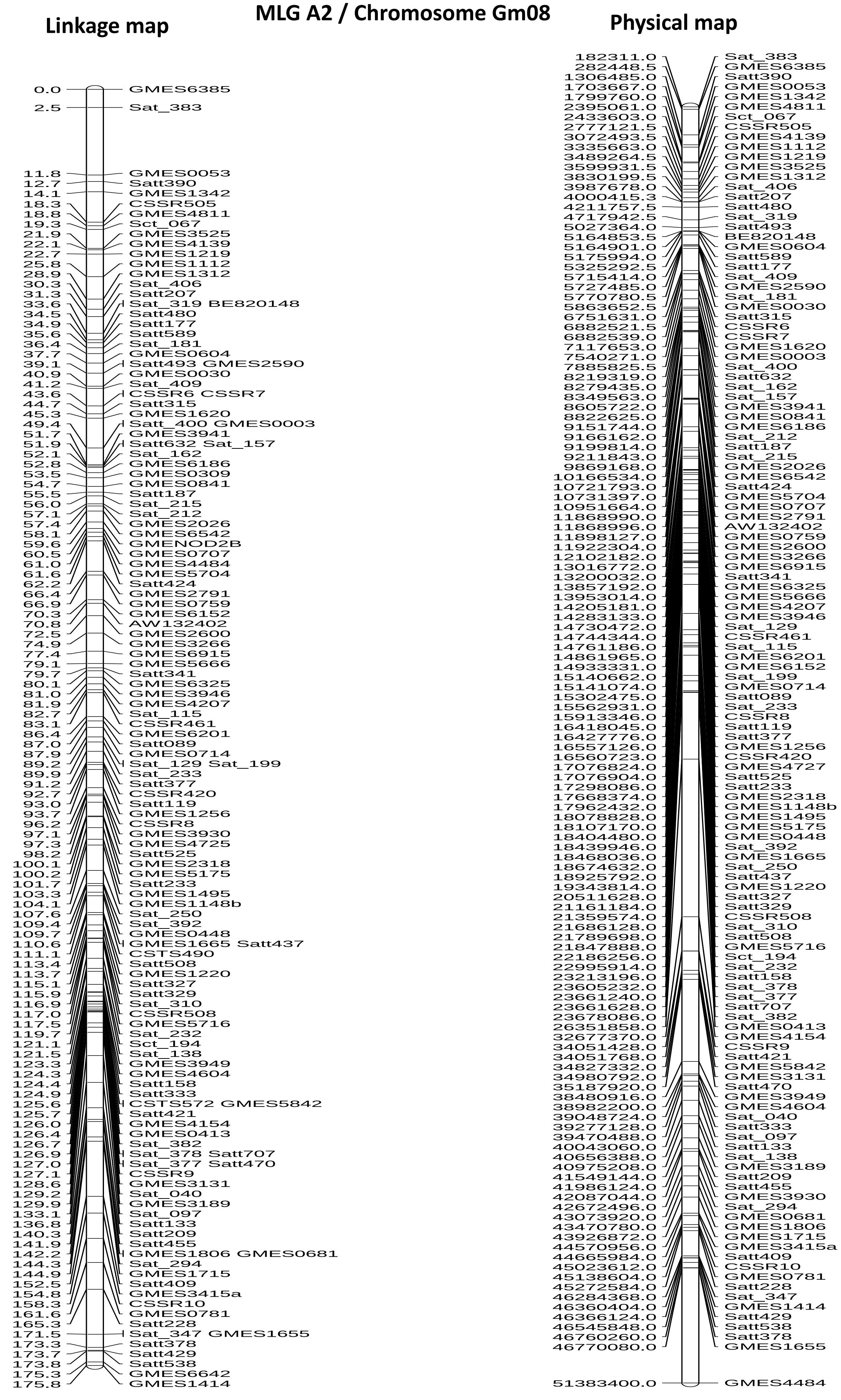

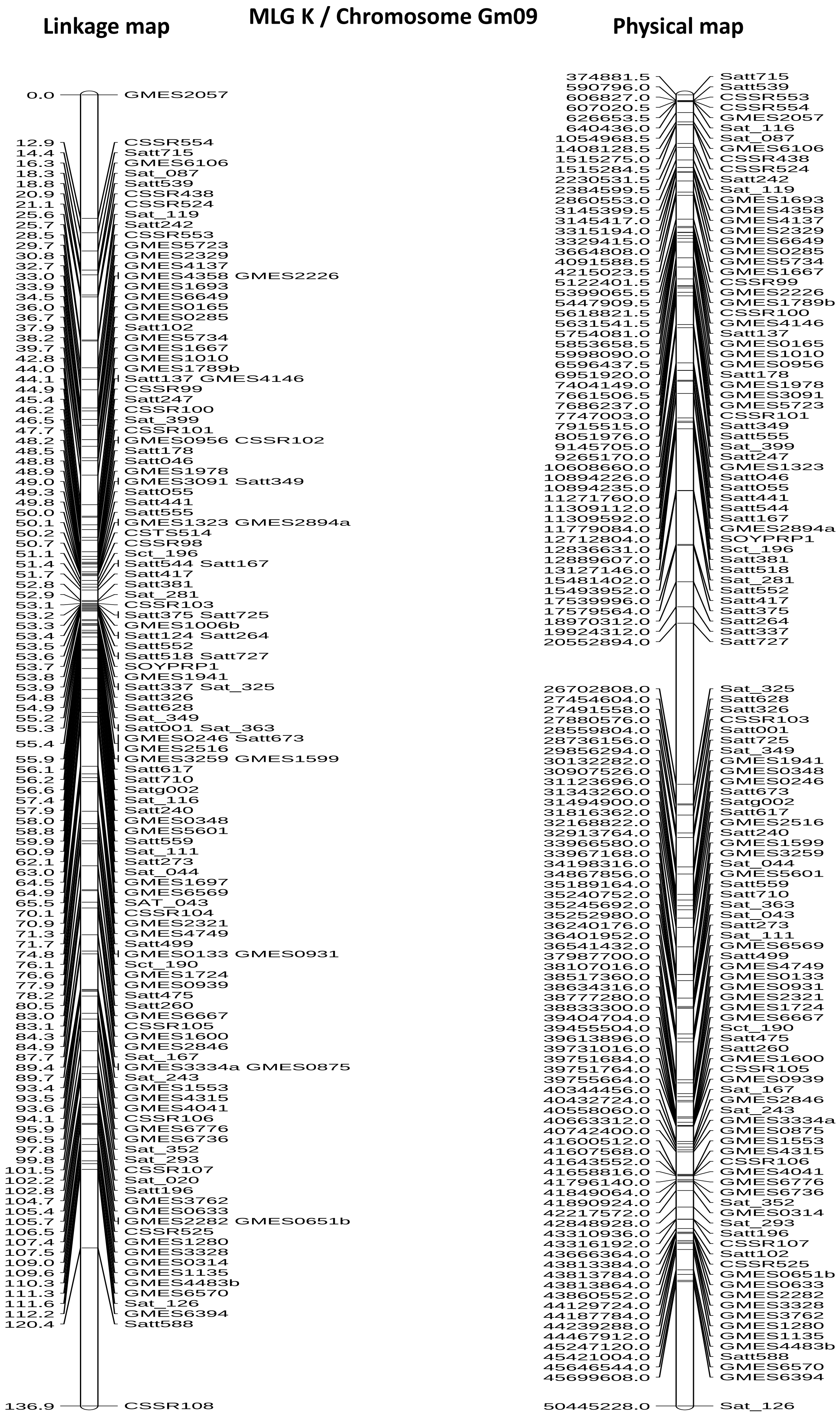
MLG K / Chromosome Gm09
Linkage map
0.0 GMES2057
12.9 CSSR554
14.4 Satt715
16.3 GMES6106
18.3 Sat_087
18.8 Satt539
20.9 CSSR438
21.1 CSSR524
25.6 Sat_119
25.7 Satt242
28.5 CSSR553
29.7 GMES5723
30.8 GMES2329
32.7 GMES4137
33.0 GMES4358 GMES2226
33.9 GMES1693
34.5 GMES6649
36.0 GMES0165
36.7 GMES0285
37.9 Satt102
38.2 GMES5734
39.7 GMES1667
42.8 GMES1010
44.0 GMES1789b
44.1 Satt137 GMES4146
44.9 CSSR99
45.4 Satt247
46.2 CSSR100
46.5 Sat_399
47.7 CSSR101
48.2 GMES0956 CSSR102
48.5 Satt178
48.8 Satt046
48.9 GMES1978
49.0 GMES3091 Satt349
49.3 Satt055
49.8 Satt441
50.0 Satt555
50.1 GMES1323 GMES2894a
50.2 CSTS514
50.7 CSSR98
51.1 Sct_196
51.4 Satt544 Satt167
51.7 Satt417
52.8 Satt381
52.9 Sat_281
53.1 CSSR103
53.2 Satt375 Satt725
53.3 GMES1006b
53.4 Satt124 Satt264
53.5 Satt552
53.6 Satt518 Satt727
53.7 SOYPRP1
53.8 GMES1941
53.9 Satt337 Sat_325
54.8 Satt326
54.9 Satt628
55.2 Sat_349
55.3 Satt001 Sat_363
55.4 GMES0246 Satt673
GMES2516
55.9 GMES3259 GMES1599
56.1 Satt617
56.2 Satt710
56.6 Satg002
57.4 Sat_116
57.9 Satt240
58.0 GMES0348
58.8 GMES5601
59.9 Satt559
60.9 Sat_111
62.1 Satt273
63.0 Sat_044
64.5 GMES1697
64.9 GMES6569
65.5 SAT_043
70.1 CSSR104
70.9 GMES2321
71.3 GMES4749
71.7 Satt499
74.8 GMES0133 GMES0931
76.1 Sct_190
76.6 GMES1724
77.9 GMES0939
78.2 Satt475
80.5 Satt260
83.0 GMES6667
83.1 CSSR105
84.3 GMES1600
84.9 GMES2846
87.7 Sat_167
89.4 GMES3334a GMES0875
89.7 Sat_243
93.4 GMES1553
93.5 GMES4315
93.6 GMES4041
94.1 CSSR106
95.9 GMES6776
96.5 GMES6736
97.8 Sat_352
99.8 Sat_293
101.5 CSSR107
102.2 Sat_020
102.8 Satt196
104.7 GMES3762
105.4 GMES0633
105.7 GMES2282 GMES0651b
106.5 CSSR525
107.4 GMES1280
107.5 GMES3328
109.0 GMES0314
109.6 GMES1135
110.3 GMES4483b
111.3 GMES6570
111.6 Sat_126
112.2 GMES6394
120.4 Satt588
136.9 CSSR108
Physical map
374881.5 Satt715
590796.0 Satt539
606827.0 CSSR553
607020.5 CSSR554
626653.5 GMES2057
640436.0 Sat_116
1054968.5 Sat_087
1408128.5 GMES6106
1515275.0 CSSR438
1515284.5 CSSR524
2230531.5 Satt242
2384599.5 Sat_119
2860553.0 GMES1693
3145399.5 GMES4358
3145417.0 GMES4137
3315194.0 GMES2329
3329415.0 GMES6649
3664808.0 GMES0285
4091588.5 GMES5734
4215023.5 GMES1667
5122401.5 CSSR99
5399065.5 GMES2226
5447909.5 GMES1789b
5618821.5 CSSR100
5631541.5 GMES4146
5754081.0 Satt137
5853658.5 GMES0165
5998090.0 GMES1010
6596437.5 GMES0956
6951920.0 Satt178
7404149.0 GMES1978
7661506.5 GMES3091
7686237.0 GMES5723
7747003.0 CSSR101
7915515.0 Satt349
8051976.0 Satt555
9145705.0 Sat_399
9265170.0 Satt247
10608660.0 GMES1323
10894226.0 Satt046
10894235.0 Satt055
11271760.0 Satt441
11309112.0 Satt544
11309592.0 Satt167
11779084.0 GMES2894a
12712804.0 SOYPRP1
12836631.0 Sct_196
12889607.0 Satt381
13127146.0 Satt518
15481402.0 Sat_281
15493952.0 Satt552
17539996.0 Satt417
17579564.0 Satt375
18970312.0 Satt264
19924312.0 Satt337
20552894.0 Satt727
26702808.0 Sat_325
27454604.0 Satt628
27491558.0 Satt326
27880576.0 CSSR103
28559804.0 Satt001
28736156.0 Satt725
29856294.0 Sat_349
30132282.0 GMES1941
30907526.0 GMES0348
31123696.0 GMES0246
31343260.0 Satt673
31494900.0 Satg002
31816362.0 Satt617
32168822.0 GMES2516
32913764.0 Satt240
33966580.0 GMES1599
33967168.0 GMES3259
34198316.0 Sat_044
34867856.0 GMES5601
35189164.0 Satt559
35240752.0 Satt710
35245692.0 Sat_363
35252980.0 Sat_043
36240176.0 Satt273
36401952.0 Sat_111
36541432.0 GMES6569
37987700.0 Satt499
38107016.0 GMES4749
38517360.0 GMES0133
38634316.0 GMES0931
38777280.0 GMES2321
38833300.0 GMES1724
39404704.0 GMES6667
39455504.0 Sct_190
39613896.0 Satt475
39731016.0 Satt260
39751684.0 GMES1600
39751764.0 CSSR105
39755664.0 GMES0939
40344456.0 Sat_167
40432724.0 GMES2846
40558060.0 Sat_243
40663312.0 GMES3334a
40742400.0 GMES0875
41600512.0 GMES1553
41607568.0 GMES4315
41643552.0 CSSR106
41658816.0 GMES4041
41796140.0 GMES6776
41849064.0 GMES6736
41890924.0 Sat_352
42217572.0 GMES0314
42848928.0 Sat_293
43310936.0 Satt196
43316192.0 CSSR107
43666364.0 Satt102
43813384.0 CSSR525
43813784.0 GMES0651b
43813864.0 GMES0633
43860552.0 GMES2282
44129724.0 GMES3328
44187784.0 GMES3762
44239288.0 GMES1280
44467912.0 GMES1135
45247120.0 GMES4483b
45421004.0 Satt588
45646544.0 GMES6570
45699608.0 GMES6394
50445228.0 Sat_126

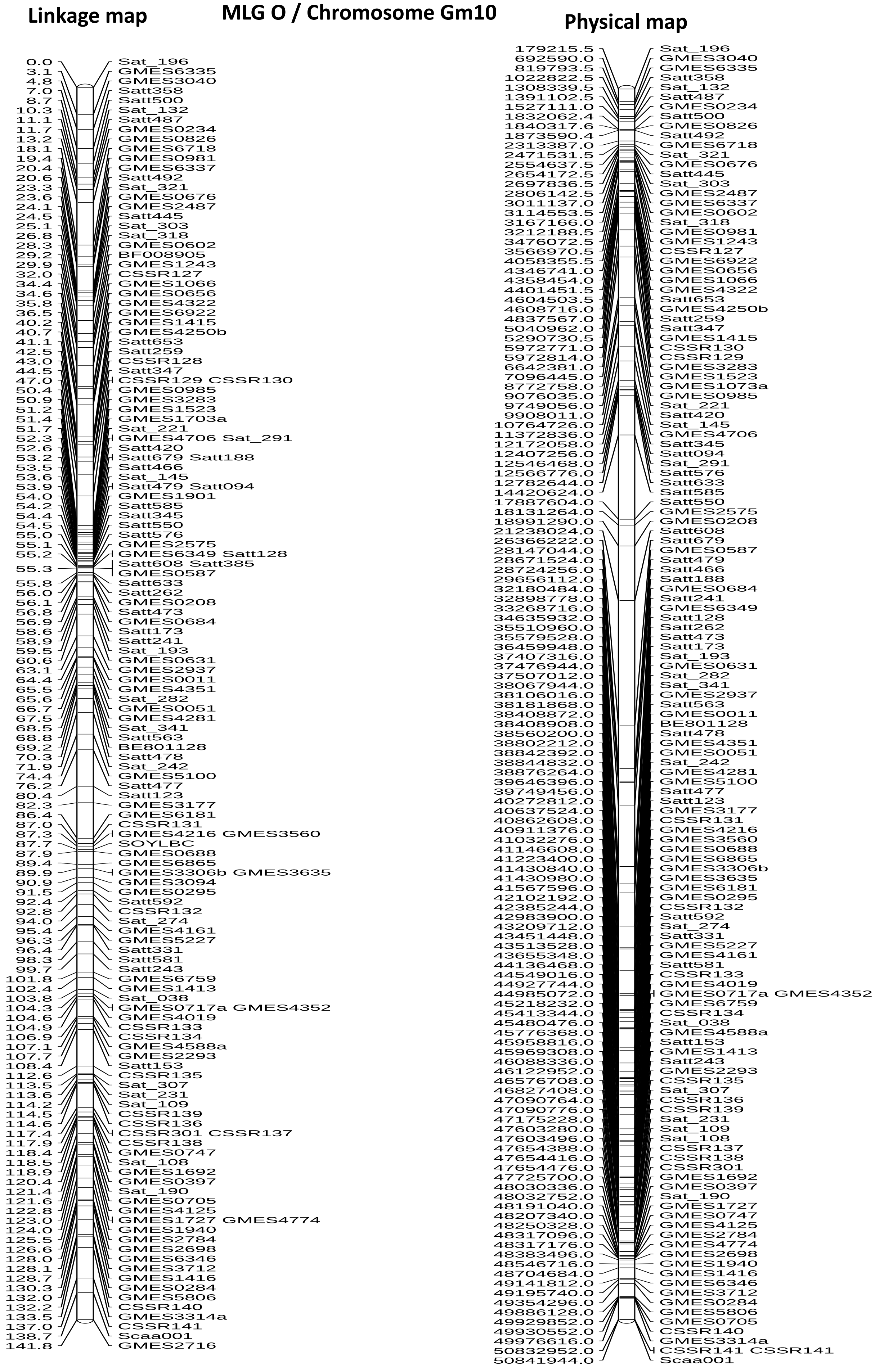
Linkage map
MLG O / Chromosome Gm10
Physical map
0.0 Sat_196
3.1 GMES6335
4.8 GMES3040
7.0 Satt358
8.7 Satt500
10.3 Sat_132
11.1 Satt487
11.7 GMES0234
13.2 GMES0826
18.1 GMES6718
19.4 GMES0981
20.4 GMES6337
20.6 Satt492
23.3 Sat_321
23.6 GMES0676
24.1 GMES2487
24.5 Satt445
25.1 Sat_303
26.8 Sat_318
28.3 GMES0602
29.2 BF008905
29.9 GMES1243
32.0 CSSR127
34.4 GMES1066
34.6 GMES0656
35.8 GMES4322
36.5 GMES6922
40.2 GMES1415
40.7 GMES4250b
41.1 Satt653
42.5 Satt259
43.0 CSSR128
44.5 Satt347
47.0 CSSR129 CSSR130
50.4 GMES0985
50.9 GMES3283
51.2 GMES1523
51.4 GMES1703a
51.7 Sat_221
52.3 GMES4706 Sat_291
52.6 Satt420
53.2 Satt679 Satt188
53.5 Satt466
53.6 Sat_145
53.9 Satt479 Satt094
54.0 GMES1901
54.2 Satt585
54.4 Satt345
54.5 Satt550
55.0 Satt576
55.1 GMES2575
55.2 GMES6349 Satt128
55.3 Satt608 Satt385
GMES0587
55.8 Satt633
56.0 Satt262
56.1 GMES0208
56.8 Satt473
56.9 GMES0684
58.6 Satt173
58.9 Satt241
59.5 Sat_193
60.6 GMES0631
63.1 GMES2937
64.4 GMES0011
65.5 GMES4351
65.6 Sat_282
66.7 GMES0051
67.5 GMES4281
68.5 Sat_341
68.8 Satt563
69.2 BE801128
70.3 Satt478
71.9 Sat_242
74.4 GMES5100
76.2 Satt477
80.4 Satt123
82.3 GMES3177
86.4 GMES6181
87.0 CSSR131
87.3 GMES4216 GMES3560
87.7 SOYLBC
87.9 GMES0688
89.4 GMES6865
89.9 GMES3306b GMES3635
90.9 GMES3094
91.5 GMES0295
92.4 Satt592
92.8 CSSR132
94.0 Sat_274
95.4 GMES4161
96.3 GMES5227
96.4 Satt331
98.3 Satt581
99.7 Satt243
101.8 GMES6759
102.4 GMES1413
103.8 Sat_038
104.3 GMES0717a GMES4352
104.6 GMES4019
104.9 CSSR133
106.9 CSSR134
107.1 GMES4588a
107.7 GMES2293
108.4 Satt153
112.6 CSSR135
113.5 Sat_307
113.6 Sat_231
114.2 Sat_109
114.5 CSSR139
114.6 CSSR136
117.4 CSSR301 CSSR137
117.9 CSSR138
118.4 GMES0747
118.5 Sat_108
118.9 GMES1692
120.4 GMES0397
121.4 Sat_190
121.6 GMES0705
122.8 GMES4125
123.0 GMES1727 GMES4774
124.0 GMES1940
125.5 GMES2784
126.6 GMES2698
128.0 GMES6346
128.1 GMES3712
128.7 GMES1416
130.3 GMES0284
132.0 GMES5806
132.2 CSSR140
133.5 GMES3314a
137.0 CSSR141
138.7 Scaa001
141.8 GMES2716
179215.5 Sat_196
692590.0 GMES3040
819793.5 GMES6335
1022822.5 Satt358
1308339.5 Sat_132
1391102.5 Satt487
1527111.0 GMES0234
1832062.4 Satt500
1840317.6 GMES0826
1873590.4 Satt492
2313387.0 GMES6718
2471531.5 Sat_321
2554637.5 GMES0676
2654172.5 Satt445
2697836.5 Sat_303
2806142.5 GMES2487
3011137.0 GMES6337
3114553.5 GMES0602
3167166.0 Sat_318
3212188.5 GMES0981
3476072.5 GMES1243
3566970.5 CSSR127
4058355.5 GMES6922
4346741.0 GMES0656
4358454.0 GMES1066
4401451.5 GMES4322
4604503.5 Satt653
4608716.0 GMES4250b
4837567.0 Satt259
5040962.0 Satt347
5290730.5 GMES1415
5972771.0 CSSR130
5972814.0 CSSR129
6642381.0 GMES3283
7096445.0 GMES1523
8772758.0 GMES1073a
9076035.0 GMES0985
9749056.0 Sat_221
9908011.0 Satt420
10764726.0 Sat_145
11372836.0 GMES4706
12172058.0 Satt345
12407256.0 Satt094
12546468.0 Sat_291
12566776.0 Satt576
12782644.0 Satt633
14420624.0 Satt585
17887604.0 Satt550
18131264.0 GMES2575
18991290.0 GMES0208
21238024.0 Satt608
26366222.0 Satt679
28147044.0 GMES0587
28671524.0 Satt479
28724256.0 Satt466
29656112.0 Satt188
32180484.0 GMES0684
32898778.0 Satt241
33268716.0 GMES6349
34635932.0 Satt128
35510960.0 Satt262
35579528.0 Satt473
36459948.0 Satt173
37407316.0 Sat_193
37476944.0 GMES0631
37507012.0 Sat_282
38067944.0 Sat_341
38106016.0 GMES2937
38181868.0 Satt563
38408872.0 GMES0011
38408908.0 BE801128
38560200.0 Satt478
38802212.0 GMES4351
38842392.0 GMES0051
38844832.0 Sat_242
38876264.0 GMES4281
39646396.0 GMES5100
39749456.0 Satt477
40272812.0 Satt123
40637524.0 GMES3177
40862608.0 CSSR131
40911376.0 GMES4216
41032276.0 GMES3560
41146608.0 GMES0688
41223400.0 GMES6865
41430840.0 GMES3306b
41430980.0 GMES3635
41567596.0 GMES6181
42102192.0 GMES0295
42385244.0 CSSR132
42983900.0 Satt592
43209712.0 Sat_274
43451448.0 Satt331
43513528.0 GMES5227
43655348.0 GMES4161
44136468.0 Satt581
44549016.0 CSSR133
44927744.0 GMES4019
44985072.0 GMES0717a GMES4352
45218232.0 GMES6759
45413344.0 CSSR134
45480476.0 Sat_038
45776368.0 GMES4588a
45958816.0 Satt153
45969308.0 GMES1413
46088336.0 Satt243
46122952.0 GMES2293
46576708.0 CSSR135
46827408.0 Sat_307
47090764.0 CSSR136
47090776.0 CSSR139
47175228.0 Sat_231
47603280.0 Sat_109
47603496.0 Sat_108
47654388.0 CSSR137
47654416.0 CSSR138
47654476.0 CSSR301
47725700.0 GMES1692
48030336.0 GMES0397
48032752.0 Sat_190
48191040.0 GMES1727
48207340.0 GMES0747
48250328.0 GMES4125
48317096.0 GMES2784
48317176.0 GMES4774
48383496.0 GMES2698
48546716.0 GMES1940
48704684.0 GMES1416
49141812.0 GMES6346
49195740.0 GMES3712
49354296.0 GMES0284
49886128.0 GMES5806
49929852.0 GMES0705
49930552.0 CSSR140
49976616.0 GMES3314a
50832952.0 CSSR141 CSSR141
50841944.0 Scaa001

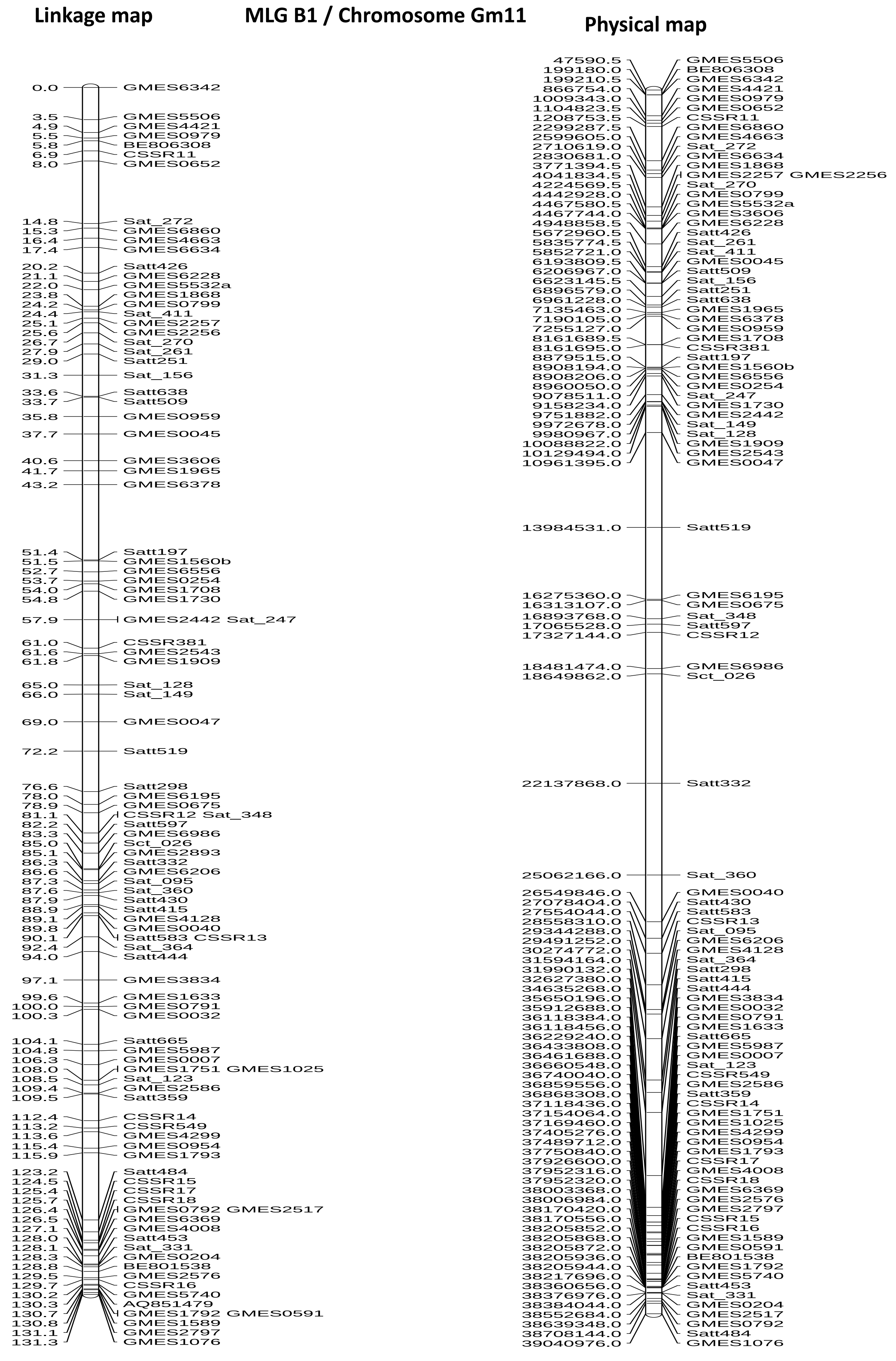
Linkage map
MLG B1 / Chromosome Gm11
Physical map
0.0 GMES6342
3.5 GMES5506
4.9 GMES4421
5.5 GMES0979
5.8 BE806308
6.9 CSSR11
8.0 GMES0652
14.8 Sat_272
15.3 GMES6860
16.4 GMES4663
17.4 GMES6634
20.2 Satt426
21.1 GMES6228
22.0 GMES5532a
23.8 GMES1868
24.2 GMES0799
24.4 Sat_411
25.1 GMES2257
25.6 GMES2256
26.7 Sat_270
27.9 Sat_261
29.0 Satt251
31.3 Sat_156
33.6 Satt638
33.7 Satt509
35.8 GMES0959
37.7 GMES0045
40.6 GMES3606
41.7 GMES1965
43.2 GMES6378
51.4 Satt197
51.5 GMES1560b
52.7 GMES6556
53.7 GMES0254
54.0 GMES1708
54.8 GMES1730
57.9 GMES2442 Sat_247
61.0 CSSR381
61.6 GMES2543
61.8 GMES1909
65.0 Sat_128
66.0 Sat_149
69.0 GMES0047
72.2 Satt519
76.6 Satt298
78.0 GMES6195
78.9 GMES0675
81.1 CSSR12 Sat_348
82.2 Satt597
83.3 GMES6986
85.0 Sct_026
85.1 GMES2893
86.3 Satt332
86.6 GMES6206
87.3 Sat_095
87.6 Sat_360
87.9 Satt430
88.9 Satt415
89.1 GMES4128
89.8 GMES0040
90.1 Satt583 CSSR13
92.4 Sat_364
94.0 Satt444
97.1 GMES3834
99.6 GMES1633
100.0 GMES0791
100.3 GMES0032
104.1 Satt665
104.8 GMES5987
106.3 GMES0007
108.0 GMES1751 GMES1025
108.5 Sat_123
109.4 GMES2586
109.5 Satt359
112.4 CSSR14
113.2 CSSR549
113.6 GMES4299
115.4 GMES0954
115.9 GMES1793
123.2 Satt484
124.5 CSSR15
125.4 CSSR17
125.7 CSSR18
126.4 GMES0792 GMES2517
126.5 GMES6369
127.1 GMES4008
128.0 Satt453
128.1 Sat_331
128.3 GMES0204
128.8 BE801538
129.5 GMES2576
129.7 CSSR16
130.2 GMES5740
130.3 AQ851479
130.7 GMES1792 GMES0591
130.8 GMES1589
131.1 GMES2797
131.3 GMES1076
47590.5 GMES5506
199180.0 BE806308
199210.5 GMES6342
866754.0 GMES4421
1009343.0 GMES0979
1104823.5 GMES0652
1208753.5 CSSR11
2299287.5 GMES6860
2599605.0 GMES4663
2710619.0 Sat_272
2830681.0 GMES6634
3771394.5 GMES1868
4041834.5 GMES2257 GMES2256
4224569.5 Sat_270
4442928.0 GMES0799
4467580.5 GMES5532a
4467744.0 GMES3606
4948858.5 GMES6228
5672960.5 Satt426
5835774.5 Sat_261
5852721.0 Sat_411
6193809.5 GMES0045
6206967.0 Satt509
6623145.5 Sat_156
6896579.0 Satt251
6961228.0 Satt638
7135463.0 GMES1965
7190105.0 GMES6378
7255127.0 GMES0959
8161689.5 GMES1708
8161695.0 CSSR381
8879515.0 Satt197
8908194.0 GMES1560b
8908206.0 GMES6556
8960050.0 GMES0254
9078511.0 Sat_247
9158234.0 GMES1730
9751882.0 GMES2442
9972678.0 Sat_149
9980967.0 Sat_128
10088822.0 GMES1909
10129494.0 GMES2543
10961395.0 GMES0047
13984531.0 Satt519
16275360.0 GMES6195
16313107.0 GMES0675
16893768.0 Sat_348
17065528.0 Satt597
17327144.0 CSSR12
18481474.0 GMES6986
18649862.0 Sct_026
22137868.0 Satt332
25062166.0 Sat_360
26549846.0 GMES0040
27078404.0 Satt430
27554044.0 Satt583
28558310.0 CSSR13
29344288.0 Sat_095
29491252.0 GMES6206
30274772.0 GMES4128
31594164.0 Sat_364
31990132.0 Satt298
32627380.0 Satt415
34635268.0 Satt444
35650196.0 GMES3834
35912688.0 GMES0032
36118384.0 GMES0791
36118456.0 GMES1633
36229240.0 Satt665
36433808.0 GMES5987
36461688.0 GMES0007
36660548.0 Sat_123
36740040.0 CSSR549
36859556.0 GMES2586
36868308.0 Satt359
37118436.0 CSSR14
37154064.0 GMES1751
37169460.0 GMES1025
37405276.0 GMES4299
37489712.0 GMES0954
37750840.0 GMES1793
37926600.0 CSSR17
37952316.0 GMES4008
37952320.0 CSSR18
38003368.0 GMES6369
38006984.0 GMES2576
38170420.0 GMES2797
38170556.0 CSSR15
38205852.0 CSSR16
38205868.0 GMES1589
38205872.0 GMES0591
38205936.0 BE801538
38205944.0 GMES1792
38217696.0 GMES5740
38360656.0 Satt453
38376976.0 Sat_331
38384044.0 GMES0204
38552684.0 GMES2517
38639348.0 GMES0792
38708144.0 Satt484
39040976.0 GMES1076

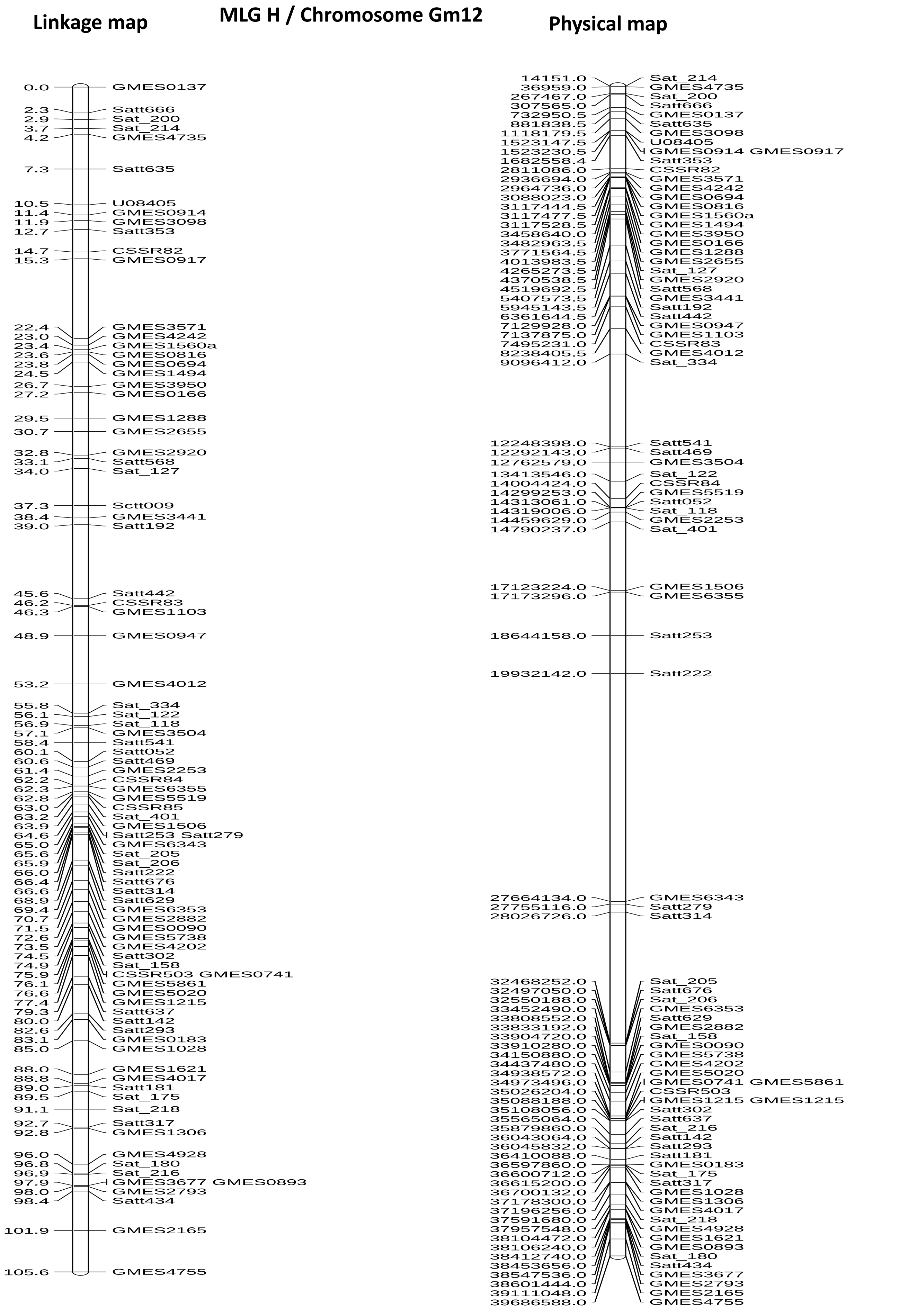
MLG H / Chromosome Gm12
Linkage map
0.0 GMES0137
2.3 Satt666
2.9 Sat_200
3.7 Sat_214
4.2 GMES4735
7.3 Satt635
10.5 U08405
11.4 GMES0914
11.9 GMES3098
12.7 Satt353
14.7 CSSR82
15.3 GMES0917
22.4 GMES3571
23.0 GMES4242
23.4 GMES1560a
23.6 GMES0816
23.8 GMES0694
24.5 GMES1494
26.7 GMES3950
27.2 GMES0166
29.5 GMES1288
30.7 GMES2655
32.8 GMES2920
33.1 Satt568
34.0 Sat_127
37.3 Sctt009
38.4 GMES3441
39.0 Satt192
45.6 Satt442
46.2 CSSR83
46.3 GMES1103
48.9 GMES0947
53.2 GMES4012
55.8 Sat_334
56.1 Sat_122
56.9 Sat_118
57.1 GMES3504
58.4 Satt541
60.1 Satt052
60.6 Satt469
61.4 GMES2253
62.2 CSSR84
62.3 GMES6355
62.8 GMES5519
63.0 CSSR85
63.2 Sat_401
63.9 GMES1506
64.6 Satt253 Satt279
65.0 GMES6343
65.6 Sat_205
65.9 Sat_206
66.0 Satt222
66.4 Satt676
66.6 Satt314
68.9 Satt629
69.4 GMES6353
70.7 GMES2882
71.5 GMES0090
72.6 GMES5738
73.5 GMES4202
74.5 Satt302
74.9 Sat_158
75.9 CSSR503 GMES0741
76.1 GMES5861
76.6 GMES5020
77.4 GMES1215
79.3 Satt637
80.0 Satt142
82.6 Satt293
83.1 GMES0183
85.0 GMES1028
88.0 GMES1621
88.8 GMES4017
89.0 Satt181
89.5 Sat_175
91.1 Sat_218
92.7 Satt317
92.8 GMES1306
96.0 GMES4928
96.8 Sat_180
96.9 Sat_216
97.9 GMES3677 GMES0893
98.0 GMES2793
98.4 Satt434
101.9 GMES2165
105.6 GMES4755
Physical map
14151.0 Sat_214
36959.0 GMES4735
267467.0 Sat_200
307565.0 Satt666
732950.5 GMES0137
881838.5 Satt635
1118179.5 GMES3098
1523147.5 U08405
1523230.5 GMES0914 GMES0917
1682558.4 Satt353
2811086.0 CSSR82
2936694.0 GMES3571
2964736.0 GMES4242
3088023.0 GMES0694
3117444.5 GMES0816
3117477.5 GMES1560a
3117528.5 GMES1494
3458640.0 GMES3950
3482963.5 GMES0166
3771564.5 GMES1288
4013983.5 GMES2655
4265273.5 Sat_127
4370538.5 GMES2920
4519692.5 Satt568
5407573.5 GMES3441
5945143.5 Satt192
6361644.5 Satt442
7129928.0 GMES0947
7137875.0 GMES1103
7495231.0 CSSR83
8238405.5 GMES4012
9096412.0 Sat_334
12248398.0 Satt541
12292143.0 Satt469
12762579.0 GMES3504
13413546.0 Sat_122
14004424.0 CSSR84
14299253.0 GMES5519
14313061.0 Satt052
14319006.0 Sat_118
14459629.0 GMES2253
14790237.0 Sat_401
17123224.0 GMES1506
17173296.0 GMES6355
18644158.0 Satt253
19932142.0 Satt222
27664134.0 GMES6343
27755116.0 Satt279
28026726.0 Satt314
32468252.0 Sat_205
32497050.0 Satt676
32550188.0 Sat_206
33452490.0 GMES6353
33808552.0 Satt629
33833192.0 GMES2882
33904720.0 Sat_158
33910280.0 GMES0090
34150880.0 GMES5738
34437480.0 GMES4202
34938572.0 GMES5020
34973496.0 GMES0741 GMES5861
35026204.0 CSSR503
35088188.0 GMES1215 GMES1215
35108056.0 Satt302
35565064.0 Satt637
35879860.0 Sat_216
36043064.0 Satt142
36045832.0 Satt293
36410088.0 Satt181
36597860.0 GMES0183
36600712.0 Sat_175
36615200.0 Satt317
36700132.0 GMES1028
37178300.0 GMES1306
37196256.0 GMES4017
37591680.0 Sat_218
37957548.0 GMES4928
38104472.0 GMES1621
38106240.0 GMES0893
38412740.0 Sat_180
38453656.0 Satt434
38547536.0 GMES3677
38601444.0 GMES2793
39111048.0 GMES2165
39686588.0 GMES4755

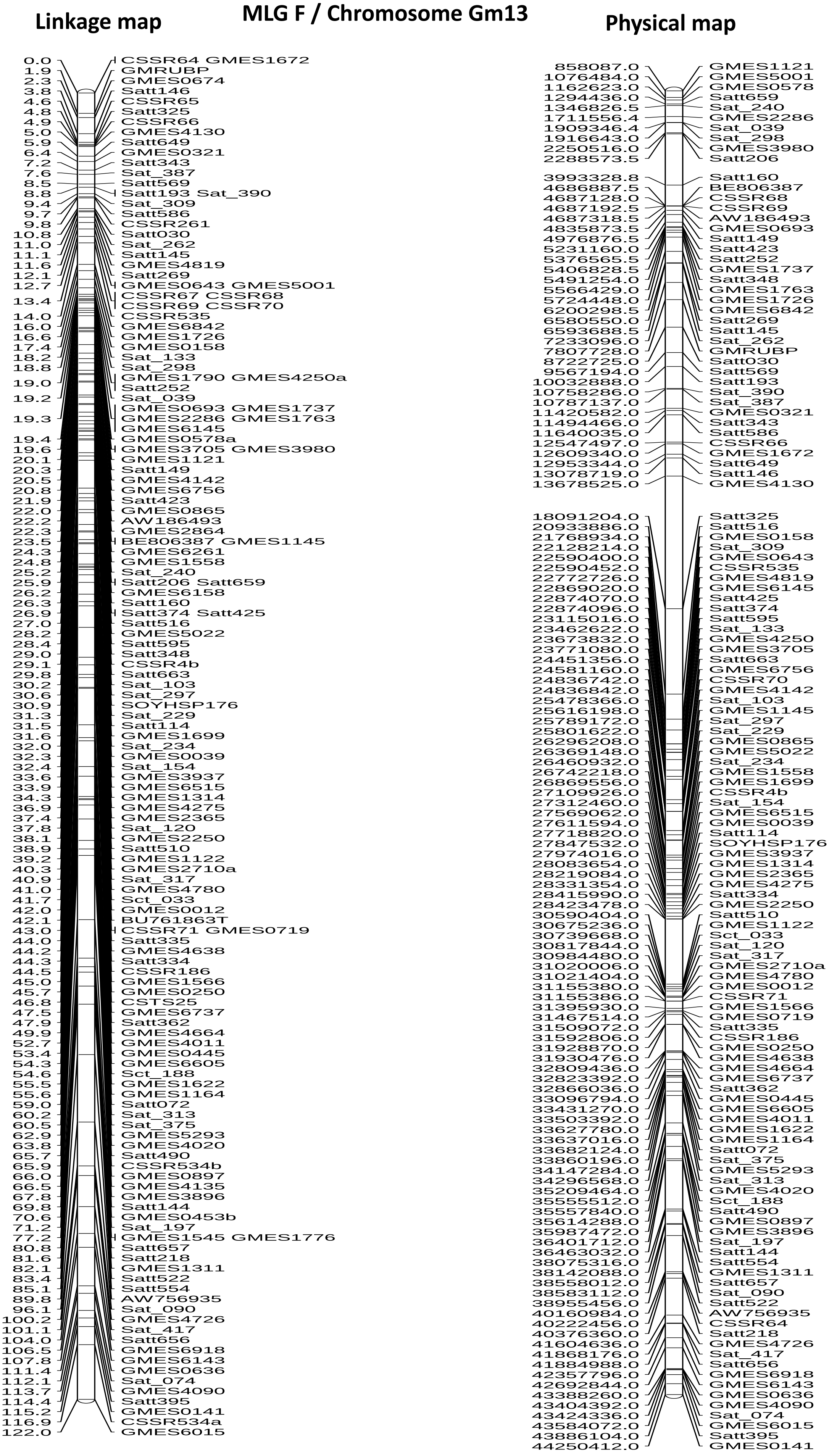
MLG F / Chromosome Gm13
Linkage map
0.0 CSSR64 GMES1672
1.9 GMRUBP
2.3 GMES0674
3.8 Satt146
4.6 CSSR65
4.8 Satt325
4.9 CSSR66
5.0 GMES4130
5.9 Satt649
6.4 GMES0321
7.2 Satt343
7.6 Sat_387
8.5 Satt569
8.8 Satt193 Sat_390
9.4 Sat_309
9.7 Satt586
9.8 CSSR261
10.8 Satt030
11.0 Sat_262
11.1 Satt145
11.6 GMES4819
12.1 Satt269
12.7 GMES0643 GMES5001
13.4 CSSR67 CSSR68
CSSR69 CSSR70
14.0 CSSR535
16.0 GMES6842
16.6 GMES1726
17.4 GMES0158
18.2 Sat_133
18.8 Sat_298
19.0 GMES1790 GMES4250a
Satt252
19.2 Sat_039
19.3 GMES0693 GMES1737
GMES2286 GMES1763
GMES6145
19.4 GMES0578a
19.6 GMES3705 GMES3980
20.1 GMES1121
20.3 Satt149
20.5 GMES4142
20.8 GMES6756
21.9 Satt423
22.0 GMES0865
22.2 AW186493
22.3 GMES2864
23.5 BE806387 GMES1145
24.3 GMES6261
24.8 GMES1558
25.2 Sat_240
25.9 Satt206 Satt659
26.2 GMES6158
26.3 Satt160
26.9 Satt374 Satt425
27.0 Satt516
28.2 GMES5022
28.4 Satt595
29.0 Satt348
29.1 CSSR4b
29.8 Satt663
30.2 Sat_103
30.6 Sat_297
30.9 SOYHSP176
31.3 Sat_229
31.5 Satt114
31.6 GMES1699
32.0 Sat_234
32.3 GMES0039
32.4 Sat_154
33.6 GMES3937
33.9 GMES6515
34.3 GMES1314
36.9 GMES4275
37.4 GMES2365
37.8 Sat_120
38.1 GMES2250
38.9 Satt510
39.2 GMES1122
40.3 GMES2710a
40.9 Sat_317
41.0 GMES4780
41.7 Sct_033
42.0 GMES0012
42.1 BU761863T
43.0 CSSR71 GMES0719
44.0 Satt335
44.2 GMES4638
44.3 Satt334
44.5 CSSR186
45.0 GMES1566
45.7 GMES0250
46.8 CSTS25
47.5 GMES6737
47.9 Satt362
49.9 GMES4664
52.7 GMES4011
53.4 GMES0445
54.3 GMES6605
54.6 Sct_188
55.5 GMES1622
55.6 GMES1164
59.0 Satt072
60.2 Sat_313
60.5 Sat_375
62.9 GMES5293
63.8 GMES4020
65.7 Satt490
65.9 CSSR534b
66.0 GMES0897
66.5 GMES4135
67.8 GMES3896
69.8 Satt144
70.6 GMES0453b
71.2 Sat_197
77.2 GMES1545 GMES1776
80.8 Satt657
81.6 Satt218
82.1 GMES1311
83.4 Satt522
85.1 Satt554
89.8 AW756935
96.1 Sat_090
100.2 GMES4726
101.1 Sat_417
104.0 Satt656
106.5 GMES6918
107.8 GMES6143
111.4 GMES0636
112.1 Sat_074
113.7 GMES4090
114.4 Satt395
115.2 GMES0141
116.9 CSSR534a
122.0 GMES6015
Physical map
858087.0 GMES1121
1076484.0 GMES5001
1162623.0 GMES0578
1294436.0 Satt659
1346826.5 Sat_240
1711556.4 GMES2286
1909346.4 Sat_039
1916643.0 Sat_298
2250516.0 GMES3980
2288573.5 Satt206
3993328.8 Satt160
4686887.5 BE806387
4687128.0 CSSR68
4687192.5 CSSR69
4687318.5 AW186493
4835873.5 GMES0693
4976876.5 Satt149
5231160.0 Satt423
5376565.5 Satt252
5406828.5 GMES1737
5491254.0 Satt348
5566429.0 GMES1763
5724448.0 GMES1726
6200298.5 GMES6842
6580550.0 Satt269
6593688.5 Satt145
7233096.0 Sat_262
7807728.0 GMRUBP
8722725.0 Satt030
9567194.0 Satt569
10032888.0 Satt193
10758286.0 Sat_390
10787137.0 Sat_387
11420582.0 GMES0321
11494466.0 Satt343
11640035.0 Satt586
12547497.0 CSSR66
12609340.0 GMES1672
12953344.0 Satt649
13078719.0 Satt146
13678525.0 GMES4130
18091204.0 Satt325
20933886.0 Satt516
21768934.0 GMES0158
22128214.0 Sat_309
22590400.0 GMES0643
22590452.0 CSSR535
22772726.0 GMES4819
22869020.0 GMES6145
22874070.0 Satt425
22874096.0 Satt374
23115016.0 Satt595
23462622.0 Sat_133
23673832.0 GMES4250
23771080.0 GMES3705
24451356.0 Satt663
24581160.0 GMES6756
24836742.0 CSSR70
24836842.0 GMES4142
25478366.0 Sat_103
25616198.0 GMES1145
25789172.0 Sat_297
25801622.0 Sat_229
26296208.0 GMES0865
26369148.0 GMES5022
26460932.0 Sat_234
26742218.0 GMES1558
26869556.0 GMES1699
27109926.0 CSSR4b
27312460.0 Sat_154
27569062.0 GMES6515
27611594.0 GMES0039
27718820.0 Satt114
27847532.0 SOYHSP176
27974016.0 GMES3937
28083654.0 GMES1314
28219084.0 GMES2365
28331354.0 GMES4275
28415990.0 Satt334
28423478.0 GMES2250
30590404.0 Satt510
30675236.0 GMES1122
30739668.0 Sct_033
30817844.0 Sat_120
30984480.0 Sat_317
31020006.0 GMES2710a
31021404.0 GMES4780
31155380.0 GMES0012
31155386.0 CSSR71
31395930.0 GMES1566
31467514.0 GMES0719
31509072.0 Satt335
31592806.0 CSSR186
31928870.0 GMES0250
31930476.0 GMES4638
32809436.0 GMES4664
32823392.0 GMES6737
32866036.0 Satt362
33096794.0 GMES0445
33431270.0 GMES6605
33503392.0 GMES4011
33627780.0 GMES1622
33637016.0 GMES1164
33682124.0 Satt072
33860196.0 Sat_375
34147284.0 GMES5293
34296568.0 Sat_313
35209464.0 GMES4020
35555512.0 Sct_188
35557840.0 Satt490
35614288.0 GMES0897
35987472.0 GMES3896
36401712.0 Sat_197
36463032.0 Satt144
38075316.0 Satt554
38142088.0 GMES1311
38558012.0 Satt657
38583112.0 Sat_090
38955456.0 Satt522
40160984.0 AW756935
40222456.0 CSSR64
40376360.0 Satt218
41604636.0 GMES4726
41868176.0 Sat_417
41884988.0 Satt656
42357796.0 GMES6918
42692844.0 GMES6143
43388260.0 GMES0636
43404392.0 GMES4090
43424336.0 Sat_074
43584072.0 GMES6015
43886104.0 Satt395
44250412.0 GMES0141

MLG B2 / Chromosome Gm14

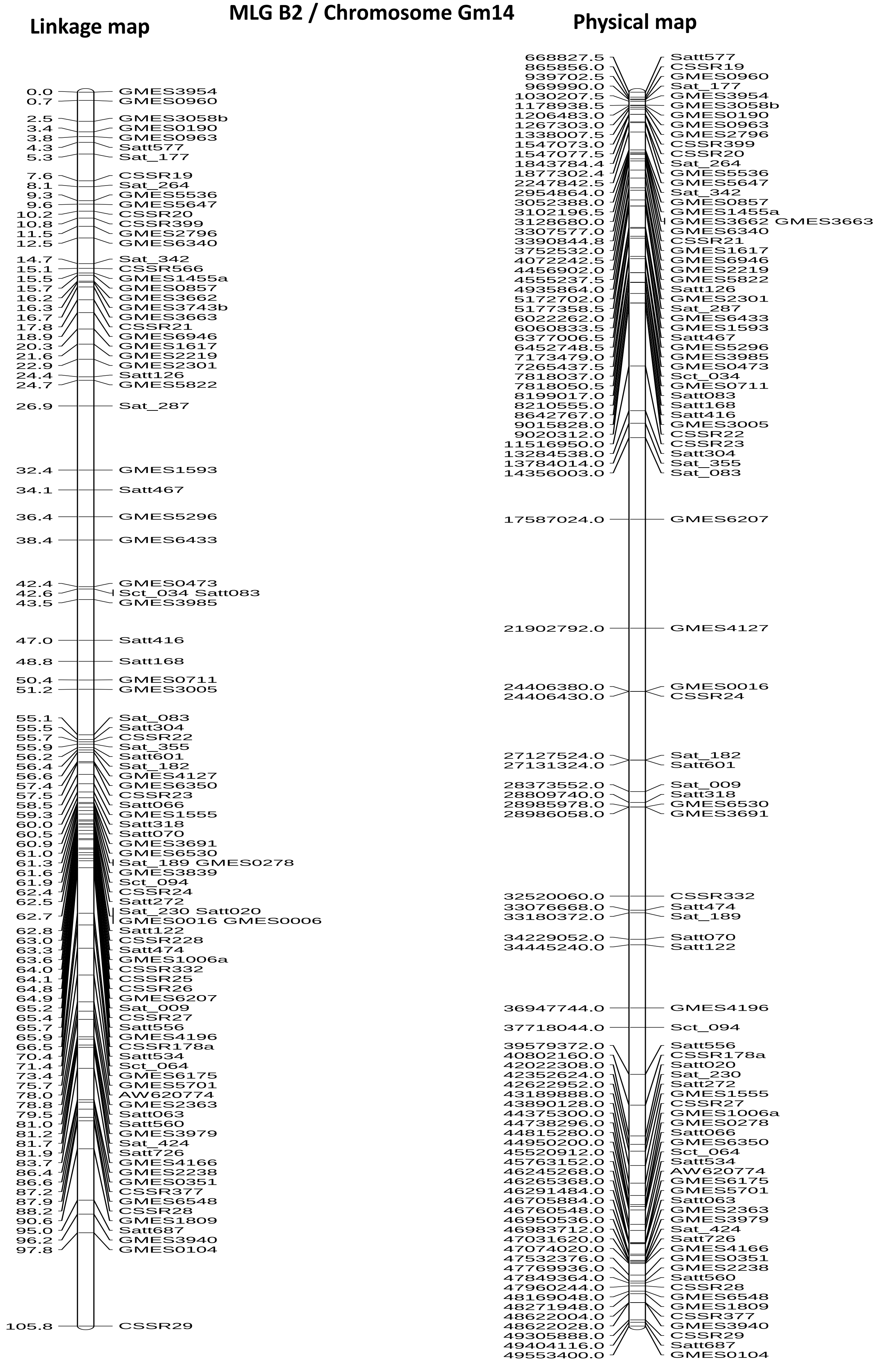

MLG E / Chromosome Gm15

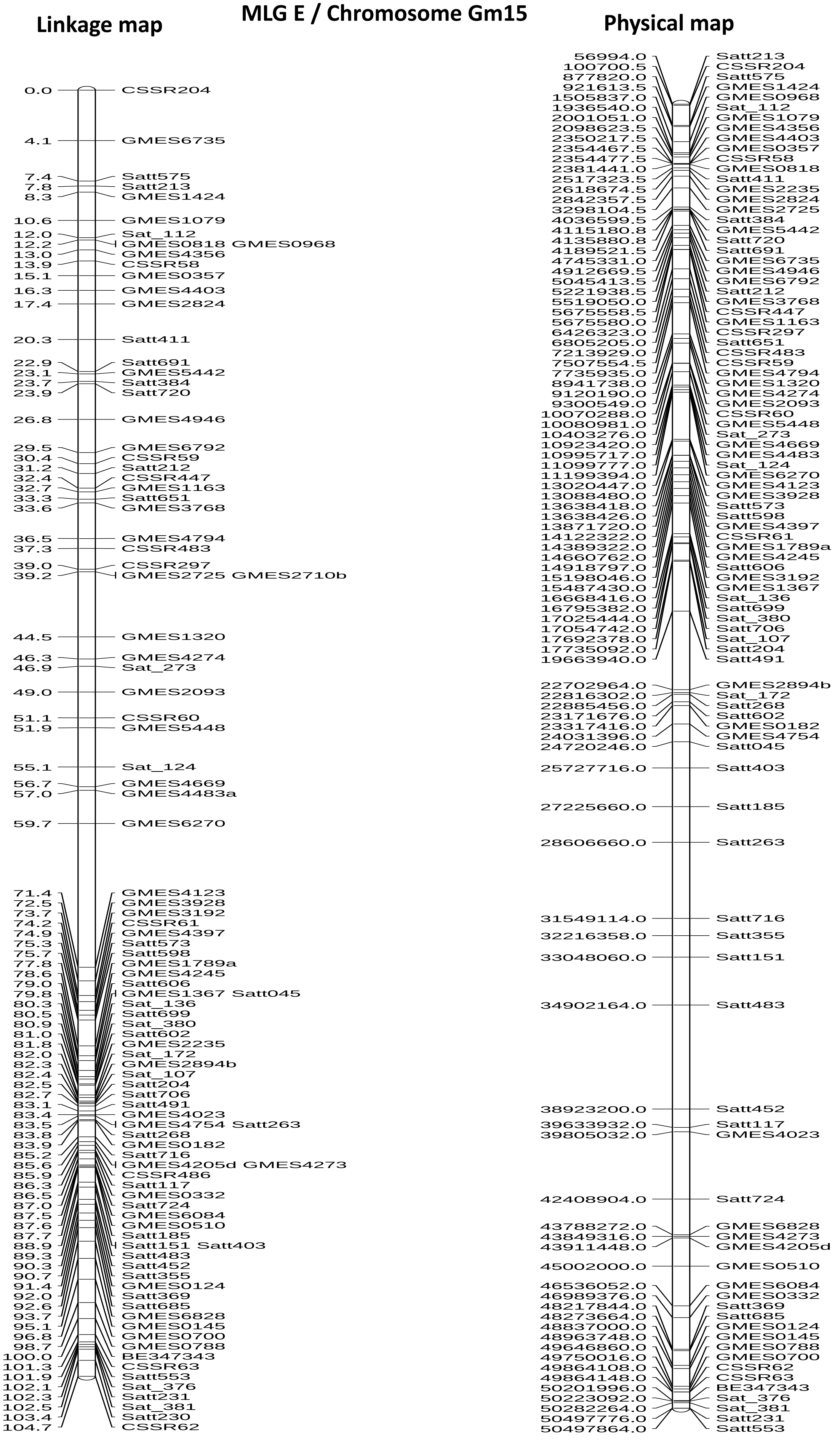

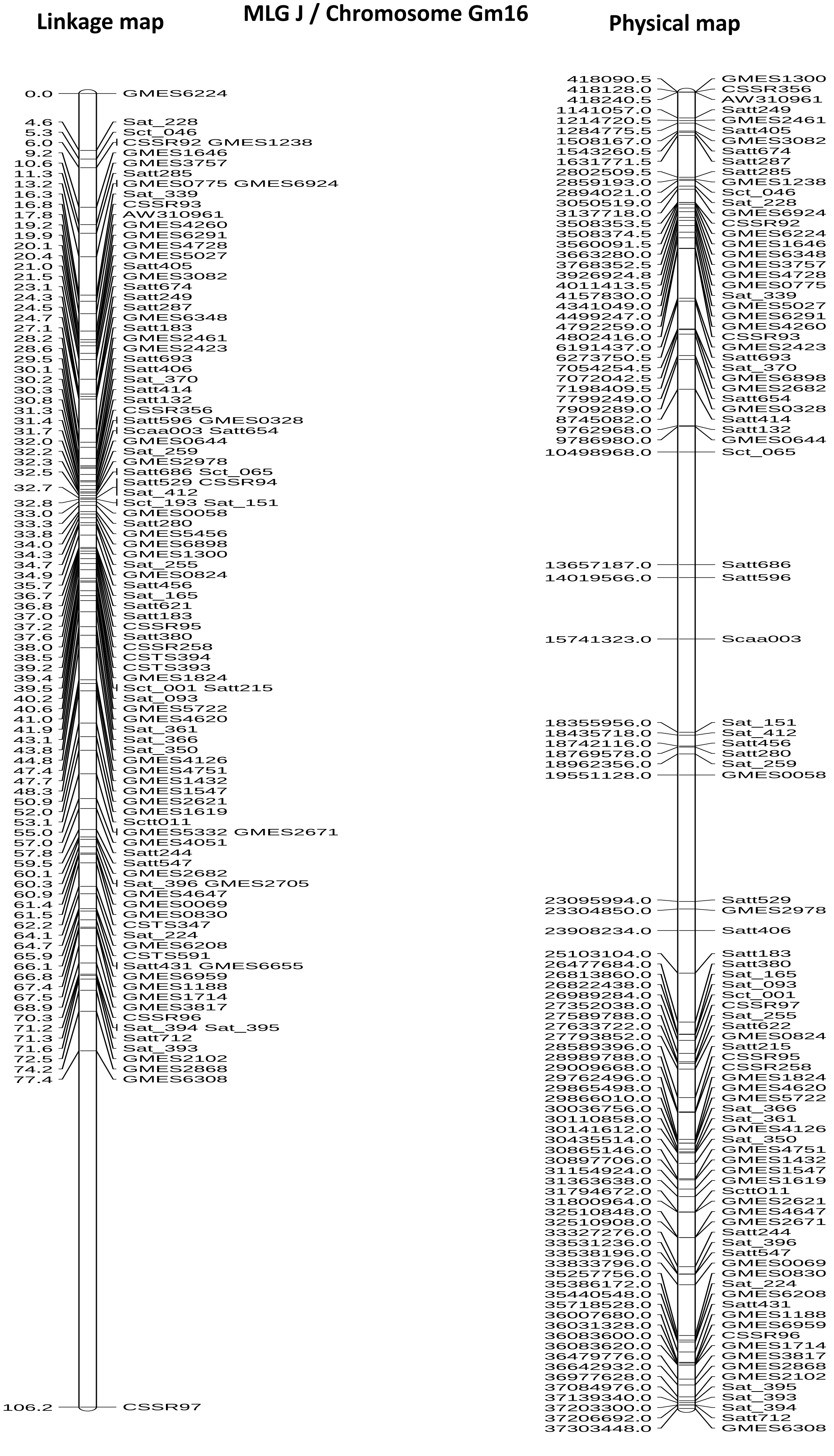
Linkage map
MLG J / Chromosome Gm16
Physical map
0.0 GMES6224
4.6 Sat_228
5.3 Sct_046
6.0 CSSR92 GMES1238
9.2 GMES1646
10.6 GMES3757
11.3 Satt285
13.2 GMES0775 GMES6924
16.3 Sat_339
16.8 CSSR93
17.8 AW310961
19.2 GMES4260
19.9 GMES6291
20.1 GMES4728
20.4 GMES5027
21.0 Satt405
21.5 GMES3082
23.1 Satt674
24.3 Satt249
24.5 Satt287
24.7 GMES6348
27.1 Satt183
28.2 GMES2461
28.6 GMES2423
29.5 Satt693
30.1 Satt406
30.2 Sat_370
30.3 Satt414
30.8 Satt132
31.3 CSSR356
31.4 Satt596 GMES0328
31.7 Scaa003 Satt654
32.0 GMES0644
32.2 Sat_259
32.3 GMES2978
32.5 Satt686 Sct_065
32.7 Satt529 CSSR94
Sat_412
32.8 Sct_193 Sat_151
33.0 GMES0058
33.3 Satt280
33.8 GMES5456
34.0 GMES6898
34.3 GMES1300
34.7 Sat_255
34.9 GMES0824
35.7 Satt456
36.7 Sat_165
36.8 Satt621
37.0 Satt183
37.2 CSSR95
37.6 Satt380
38.0 CSSR258
38.5 CSTS394
39.2 CSTS393
39.4 GMES1824
39.5 Sct_001 Satt215
40.2 Sat_093
40.6 GMES5722
41.0 GMES4620
41.9 Sat_361
43.1 Sat_366
43.8 Sat_350
44.8 GMES4126
47.4 GMES4751
47.7 GMES1432
48.3 GMES1547
50.9 GMES2621
52.0 GMES1619
53.1 Sctt011
55.0 GMES5332 GMES2671
57.0 GMES4051
57.8 Satt244
59.5 Satt547
60.1 GMES2682
60.3 Sat_396 GMES2705
60.9 GMES4647
61.4 GMES0069
61.5 GMES0830
62.2 CSTS347
64.1 Sat_224
64.7 GMES6208
65.9 CSTS591
66.1 Satt431 GMES6655
66.8 GMES6959
67.4 GMES1188
67.5 GMES1714
68.9 GMES3817
70.3 CSSR96
71.2 Sat_394 Sat_395
71.3 Satt712
71.6 Sat_393
72.5 GMES2102
74.2 GMES2868
77.4 GMES6308
106.2 CSSR97
418090.5 GMES1300
418128.0 CSSR356
418240.5 AW310961
1141057.0 Satt249
1214720.5 GMES2461
1284775.5 Satt405
1508167.0 GMES3082
1543260.5 Satt674
1631771.5 Satt287
2802509.5 Satt285
2859193.0 GMES1238
2894021.0 Sct_046
3050519.0 Sat_228
3137718.0 GMES6924
3508353.5 CSSR92
3508374.5 GMES6224
3560091.5 GMES1646
3663280.0 GMES6348
3768352.5 GMES3757
3926924.8 GMES4728
4011413.5 GMES0775
4157830.0 Sat_339
4341049.0 GMES5027
4499247.0 GMES6291
4792259.0 GMES4260
4802416.0 CSSR93
6191437.0 GMES2423
6273750.5 Satt693
7054254.5 Sat_370
7072042.5 GMES6898
7198409.5 GMES2682
7799249.0 Satt654
7909289.0 GMES0328
8745082.0 Satt414
9762968.0 Satt132
9786980.0 GMES0644
10498968.0 Sct_065
13657187.0 Satt686
14019566.0 Satt596
15741323.0 Scaa003
18355956.0 Sat_151
18435718.0 Sat_412
18742116.0 Satt456
18769578.0 Satt280
18962356.0 Sat_259
19551128.0 GMES0058
23095994.0 Satt529
23304850.0 GMES2978
23908234.0 Satt406
25103104.0 Satt183
26477684.0 Satt380
26813860.0 Sat_165
26822438.0 Sat_093
26989284.0 Sct_001
27352038.0 CSSR97
27589788.0 Sat_255
27633722.0 Satt622
27793852.0 GMES0824
28589396.0 Satt215
28989788.0 CSSR95
29009668.0 CSSR258
29762496.0 GMES1824
29865498.0 GMES4620
29866010.0 GMES5722
30036756.0 Sat_366
30110858.0 Sat_361
30141612.0 GMES4126
30435514.0 Sat_350
30865146.0 GMES4751
30897706.0 GMES1432
31154924.0 GMES1547
31363638.0 GMES1619
31794672.0 Sctt011
31800964.0 GMES2621
32510848.0 GMES4647
32510908.0 GMES2671
33327276.0 Satt244
33531236.0 Sat_396
33538196.0 Satt547
33833796.0 GMES0069
35257756.0 GMES0830
35386172.0 Sat_224
35440548.0 GMES6208
35718528.0 Satt431
36007680.0 GMES1188
36031328.0 GMES6959
36083600.0 CSSR96
36083620.0 GMES1714
36479776.0 GMES3817
36642932.0 GMES2868
36977628.0 GMES2102
37084976.0 Sat_395
37139340.0 Sat_393
37203300.0 Sat_394
37206692.0 Satt712
37303448.0 GMES6308

MLG D2 / Chromosome Gm17

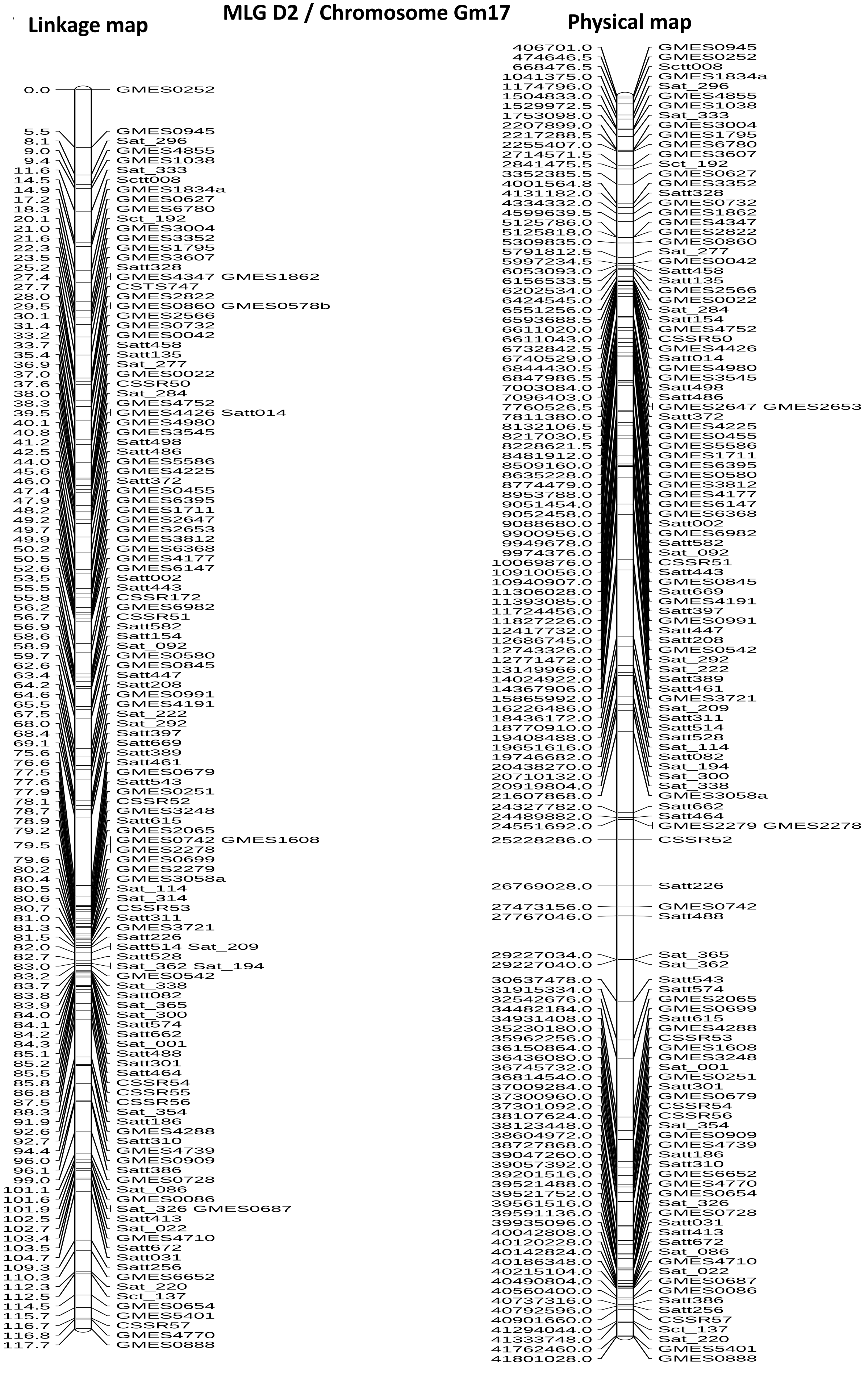

MLG G / Chromosome Gm18

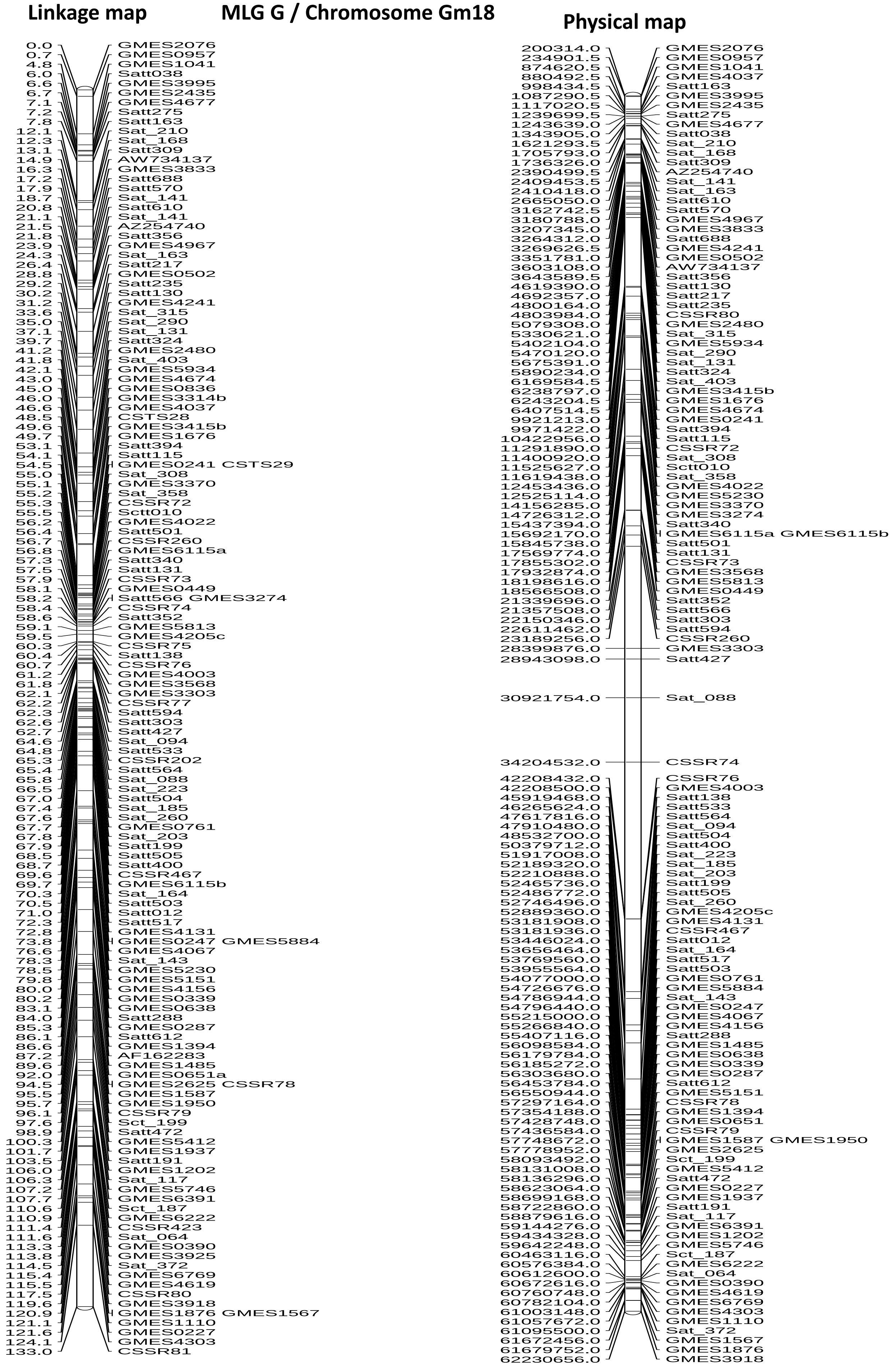

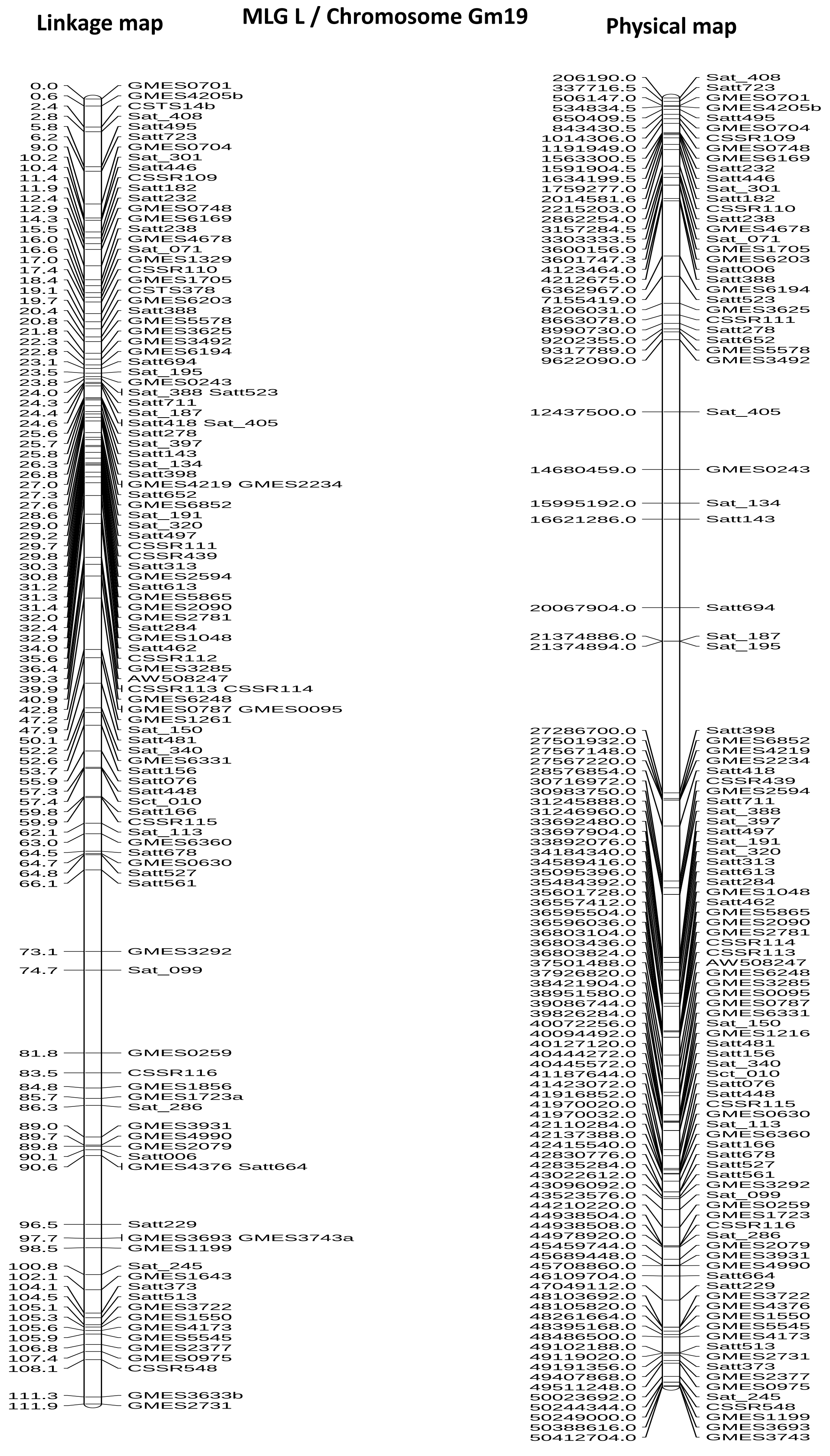
MLG L / Chromosome Gm19
Linkage map
0.0 GMES0701
0.6 GMES4205b
2.4 CSTS14b
2.8 Sat_408
5.8 Satt495
6.2 Satt723
9.0 GMES0704
10.2 Sat_301
10.4 Satt446
11.4 CSSR109
11.9 Satt182
12.4 Satt232
12.9 GMES0748
14.3 GMES6169
15.5 Satt238
16.0 GMES4678
16.6 Sat_071
17.0 GMES1329
17.4 CSSR110
18.4 GMES1705
19.1 CSTS378
19.7 GMES6203
20.4 Satt388
20.8 GMES5578
21.8 GMES3625
22.3 GMES3492
22.8 GMES6194
23.1 Satt694
23.5 Sat_195
23.8 GMES0243
24.0 Sat_388 Satt523
24.3 Satt711
24.4 Sat_187
24.6 Satt418 Sat_405
25.6 Satt278
25.7 Sat_397
25.8 Satt143
26.3 Sat_134
26.8 Satt398
27.0 GMES4219 GMES2234
27.3 Satt652
27.6 GMES6852
28.6 Sat_191
29.0 Sat_320
29.2 Satt497
29.7 CSSR111
29.8 CSSR439
30.3 Satt313
30.8 GMES2594
31.2 Satt613
31.3 GMES5865
31.4 GMES2090
32.0 GMES2781
32.4 Satt284
32.9 GMES1048
34.0 Satt462
35.6 CSSR112
36.4 GMES3285
39.3 AW508247
39.9 CSSR113 CSSR114
40.9 GMES6248
42.8 GMES0787 GMES0095
47.2 GMES1261
47.9 Sat_150
50.1 Satt481
52.2 Sat_340
52.6 GMES6331
53.7 Satt156
55.9 Satt076
57.3 Satt448
57.4 Sct_010
59.8 Satt166
59.9 CSSR115
62.1 Sat_113
63.0 GMES6360
64.5 Satt678
64.7 GMES0630
64.8 Satt527
66.1 Satt561
73.1 GMES3292
74.7 Sat_099
81.8 GMES0259
83.5 CSSR116
84.8 GMES1856
85.7 GMES1723a
86.3 Sat_286
89.0 GMES3931
89.7 GMES4990
89.8 GMES2079
90.1 Satt006
90.6 GMES4376 Satt664
96.5 Satt229
97.7 GMES3693 GMES3743a
98.5 GMES1199
100.8 Sat_245
102.1 GMES1643
104.1 Satt373
104.5 Satt513
105.1 GMES3722
105.3 GMES1550
105.6 GMES4173
105.9 GMES5545
106.8 GMES2377
107.4 GMES0975
108.1 CSSR548
111.3 GMES3633b
111.9 GMES2731
Physical map
206190.0 Sat_408
337716.5 Satt723
506147.0 GMES0701
534834.5 GMES4205b
650409.5 Satt495
843430.5 GMES0704
1014306.0 CSSR109
1191949.0 GMES0748
1563300.5 GMES6169
1591904.5 Satt232
1634199.5 Satt446
1759277.0 Sat_301
2014581.6 Satt182
2215203.0 CSSR110
2862254.0 Satt238
3157284.5 GMES4678
3303333.5 Sat_071
3600156.0 GMES1705
3601747.3 GMES6203
4123464.0 Satt006
4212675.0 Satt388
6362967.0 GMES6194
7155419.0 Satt523
8206031.0 GMES3625
8663078.0 CSSR111
8990730.0 Satt278
9202355.0 Satt652
9317789.0 GMES5578
9622090.0 GMES3492
12437500.0 Sat_405
14680459.0 GMES0243
15995192.0 Sat_134
16621286.0 Satt143
20067904.0 Satt694
21374886.0 Sat_187
21374894.0 Sat_195
27286700.0 Satt398
27501932.0 GMES6852
27567148.0 GMES4219
27567220.0 GMES2234
28576854.0 Satt418
30716972.0 CSSR439
30983750.0 GMES2594
31245888.0 Satt711
31246960.0 Sat_388
33692480.0 Sat_397
33697904.0 Satt497
33892076.0 Sat_191
34184340.0 Sat_320
34589416.0 Satt313
35095396.0 Satt613
35484392.0 Satt284
35601728.0 GMES1048
36557412.0 Satt462
36595504.0 GMES5865
36596036.0 GMES2090
36803104.0 GMES2781
36803436.0 CSSR114
36803824.0 CSSR113
37501488.0 AW508247
37926820.0 GMES6248
38421904.0 GMES3285
38951580.0 GMES0095
39086744.0 GMES0787
39826284.0 GMES6331
40072256.0 Sat_150
40094492.0 GMES1216
40127120.0 Satt481
40444272.0 Satt156
40445572.0 Sat_340
41187644.0 Sct_010
41423072.0 Satt076
41916852.0 Satt448
41970020.0 CSSR115
41970032.0 GMES0630
42110284.0 Sat_113
42137388.0 GMES6360
42415540.0 Satt166
42830776.0 Satt678
42835284.0 Satt527
43022612.0 Satt561
43096092.0 GMES3292
43523576.0 Sat_099
44210220.0 GMES0259
44938504.0 GMES1723
44938508.0 CSSR116
44978920.0 Sat_286
45459744.0 GMES2079
45689448.0 GMES3931
45708860.0 GMES4990
46109704.0 Satt664
47049112.0 Satt229
48103692.0 GMES3722
48105820.0 GMES4376
48261664.0 GMES1550
48395168.0 GMES5545
48486500.0 GMES4173
49102188.0 Satt513
49119020.0 GMES2731
49191356.0 Satt373
49407868.0 GMES2377
49511248.0 GMES0975
50023692.0 Sat_245
50244344.0 CSSR548
50249000.0 GMES1199
50388616.0 GMES3693
50412704.0 GMES3743

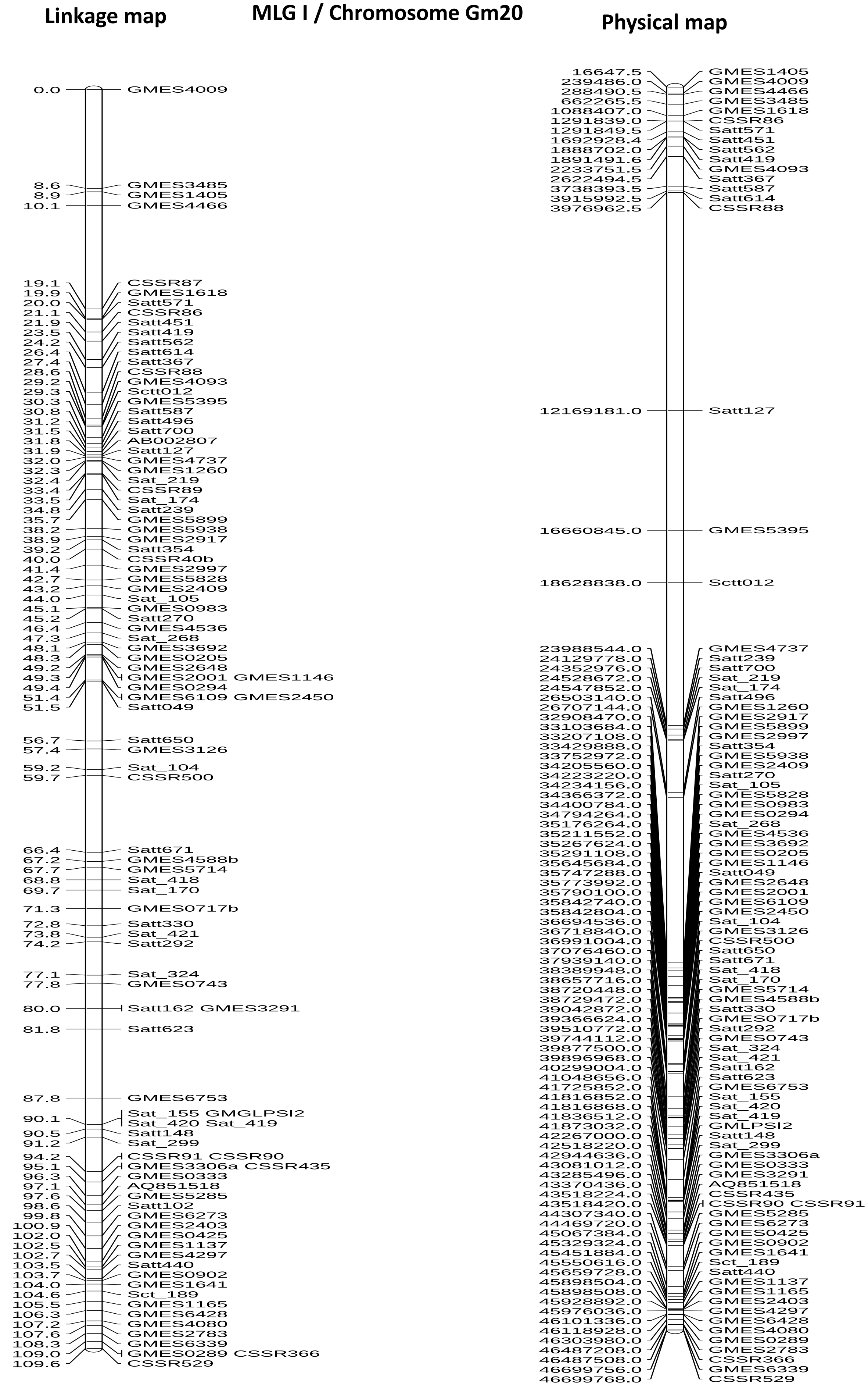
Linkage map
MLG I / Chromosome Gm20
Physical map
0.0 GMES4009
8.6 GMES3485
8.9 GMES1405
10.1 GMES4466
19.1 CSSR87
19.9 GMES1618
20.0 Satt571
21.1 CSSR86
21.9 Satt451
23.5 Satt419
24.2 Satt562
26.4 Satt614
27.4 Satt367
28.6 CSSR88
29.2 GMES4093
29.3 Sctt012
30.3 GMES5395
30.8 Satt587
31.2 Satt496
31.5 Satt700
31.8 AB002807
31.9 Satt127
32.0 GMES4737
32.3 GMES1260
32.4 Sat_219
33.4 CSSR89
33.5 Sat_174
34.8 Satt239
35.7 GMES5899
38.2 GMES5938
38.9 GMES2917
39.2 Satt354
40.0 CSSR40b
41.4 GMES2997
42.7 GMES5828
43.2 GMES2409
44.0 Sat_105
45.1 GMES0983
45.2 Satt270
46.4 GMES4536
47.3 Sat_268
48.1 GMES3692
48.3 GMES0205
49.2 GMES2648
49.3 GMES2001 GMES1146
49.4 GMES0294
51.4 GMES6109 GMES2450
51.5 Satt049
56.7 Satt650
57.4 GMES3126
59.2 Sat_104
59.7 CSSR500
66.4 Satt671
67.2 GMES4588b
67.7 GMES5714
68.8 Sat_418
69.7 Sat_170
71.3 GMES0717b
72.8 Satt330
73.8 Sat_421
74.2 Satt292
77.1 Sat_324
77.8 GMES0743
80.0 Satt162 GMES3291
81.8 Satt623
87.8 GMES6753
90.1 Sat_155 GMGLPSI2 Sat_420 Sat_419
90.5 Satt148
91.2 Sat_299
94.2 CSSR91 CSSR90
95.1 GMES3306a CSSR435
96.3 GMES0333
97.1 AQ851518
97.6 GMES5285
98.6 Satt102
99.8 GMES6273
100.9 GMES2403
102.0 GMES0425
102.5 GMES1137
102.7 GMES4297
103.5 Satt440
103.7 GMES0902
104.0 GMES1641
104.6 Sct_189
105.5 GMES1165
106.3 GMES6428
107.2 GMES4080
107.6 GMES2783
108.3 GMES6339
109.0 GMES0289 CSSR366
109.6 CSSR529
16647.5 GMES1405
239486.0 GMES4009
288490.5 GMES4466
662265.5 GMES3485
1088407.0 GMES1618
1291839.0 CSSR86
1291849.5 Satt571
1692928.4 Satt451
1888702.0 Satt562
1891491.6 Satt419
2233751.5 GMES4093
2622494.5 Satt367
3738393.5 Satt587
3915992.5 Satt614
3976962.5 CSSR88
12169181.0 Satt127
16660845.0 GMES5395
18628838.0 Sctt012
23988544.0 GMES4737
24129778.0 Satt239
24352976.0 Satt700
24528672.0 Sat_219
24547852.0 Sat_174
26503140.0 Satt496
26707144.0 GMES1260
32908470.0 GMES2917
33103684.0 GMES5899
33207108.0 GMES2997
33429888.0 Satt354
33752972.0 GMES5938
34205560.0 GMES2409
34223220.0 Satt270
34234156.0 Sat_105
34366372.0 GMES5828
34400784.0 GMES0983
34794264.0 GMES0294
35176264.0 Sat_268
35211552.0 GMES4536
35267624.0 GMES3692
35291108.0 GMES0205
35645684.0 GMES1146
35747288.0 Satt049
35773992.0 GMES2648
35790100.0 GMES2001
35842740.0 GMES6109
35842804.0 GMES2450
36694536.0 Sat_104
36718840.0 GMES3126
36991004.0 CSSR500
37076460.0 Satt650
37939140.0 Satt671
38389948.0 Sat_418
38657716.0 Sat_170
38720448.0 GMES5714
38729472.0 GMES4588b
39042872.0 Satt330
39366624.0 GMES0717b
39510772.0 Satt292
39744112.0 GMES0743
39877500.0 Sat_324
39896968.0 Sat_421
40299004.0 Satt162
41048656.0 Satt623
41725852.0 GMES6753
41816852.0 Sat_155
41816868.0 Sat_420
41836512.0 Sat_419
41873032.0 GMLPSI2
42267000.0 Satt148
42518220.0 Sat_299
42944636.0 GMES3306a
43081012.0 GMES0333
43285496.0 GMES3291
43370436.0 AQ851518
43518224.0 CSSR435
43518420.0 CSSR90 CSSR91
44307340.0 GMES5285
44469720.0 GMES6273
45067384.0 GMES0425
45329324.0 GMES0902
45451884.0 GMES1641
45550616.0 Sct_189
45659728.0 Satt440
45898504.0 GMES1137
45898508.0 GMES1165
45928892.0 GMES2403
45976036.0 GMES4297
46101336.0 GMES6428
46118928.0 GMES4080
46303980.0 GMES0289
46487208.0 GMES2783
46487508.0 CSSR366
46699756.0 GMES6339
46699768.0 CSSR529
